# Effectiveness of a family-oriented intervention for children of mentally ill parents on children’s mental health and health-related quality of life: a prospective, rater-blinded, cluster-randomized controlled multicenter trial

**DOI:** 10.1186/s13034-026-01174-6

**Published:** 2026-09-25

**Authors:** Theresa Paumen, Mareike Busmann, Anne Daubmann, Alexandra Höller, Ann-Kathrin Ozga, Reinhold Kilian, Sibylle M. Winter, Martin Lambert, Karl Wegscheider, Antonia Zapf, Silke Wiegand-Grefe

**Affiliations:** 1 Department of Psychiatry and Psychotherapy, University Medical Center Hamburg-Eppendorf, Martinistraße 52, 20246 Hamburg, Germany; 2 Institute of Medical Biometry and Epidemiology, University Medical Center Hamburg-Eppendorf, Martinistraße 52, 20246 Hamburg, Germany; 3 Department of Psychiatry II, Section: Health Economics and Health Services Research, University of Ulm, Bezirkskrankenhaus Günzburg, Lindenallee 2, 89312 Günzburg, Germany; 4 German Center for Mental Health (DZPG), Virchowweg 23, 10117 Berlin, Germany; 5 Department of Child and Adolescent Psychiatry, Psychotherapy and Psychosomatics, Charité – Universitätsmedizin Berlin, Augustenburger Platz 1, 13353 Berlin, Germany; 6 The German Center for Child and Adolescent Health (DZKJ), Study Site Hamburg, Martinistraße 52, 20246 Hamburg, Germany

**Keywords:** Transgenerational mental illness, Psychosocial care, Family therapy, Systemic therapy, Psychodynamic work with families, Psychotherapy research

## Abstract

**Background:**

Children of mentally ill parents (CHIMPS) experience a variety of negative outcomes and have an increased risk for mental illness. Nevertheless, no routine screenings or therapies for CHIMPS exist in the German healthcare system. The CHIMPS program was developed as a psychodynamic, transdiagnostic family intervention for affected children and adolescents (CA). Its effectiveness on mental health and health-related quality of life was evaluated in a prospective, two-arm, cluster-randomized, controlled, open, multicentric trial.

**Methods:**

Data from families with mentally ill parents were collected at seven sites in Germany and Switzerland at four measurement points. Group allocation was randomized. The intervention group (IG) received the CHIMPS intervention, while the control group (CG) received Treatment-As-Usual. The primary outcome was the mental health of CA with an initial psychiatric diagnosis according to the Schedule for Affective Disorders and Schizophrenia for School Aged Children, Lifetime Version (applied by blinded external raters) 18 months after baseline. Secondary outcomes comprised mental health assessed with the parent-rated Child Behavior Checklist and the self-rated Youth Self Report, and health-related quality of life assessed with the KIDSCREEN-27 (parent- and self-rated). Multilevel models were used for analysis.

**Results:**

Two hundred fifteen families (109 in IG, 106 in CG) were included in the trial. Data from 43 CA in both the IG and CG were used in the primary analysis. An odds ratio of 0.52 (95% CI: 0.25; 1.09) indicated a statistically not significant advantage of the IG over the CG. No clinically relevant advantage of the IG in any secondary outcome was found.

**Conclusions:**

The CHIMPS program will be revised to provide families with more needs-based assistance. A diagnostic evaluation, which was delivered to both IG and CG families, may have had a therapeutic effect. Future trials should place particular emphasis on improving accessibility and adherence among CHIMPS and their families, as the target group proved difficult to reach and retain. Above all, interdisciplinary structures that enable CHIMPS and their families to receive low-threshold care must be created.

*ClinicalTrials.gov* NCT0230846 (December 4, 2014).

*German Clinical Trials Register* DRKS00006806 (October 7, 2014).

**Supplementary Information:**

The online version contains supplementary material available at https://doi.org/10.1186/s13034-026-01174-6.

## Background

Mental illnesses are among the ten most common types of illness worldwide and are considered the main cause of global health-related burden [1]. In Germany, 37.9% of people with statutory health insurance were diagnosed with a mental illness in 2022 [2], and many patients are parents [3, 4]. It is estimated that 3.8 million children and adolescents (CA) in Germany live with a mentally ill parent [5].

Children of mentally ill parents (CHIMPS) often experience conflicting situations, such as feelings of shame and guilt, helplessness, insecurity, social isolation, stigmatization, and reluctance to talk about their parent’s illness [4, 6, 7]. They frequently take on responsibilities that are inappropriate for their age [4, 6]. Parenting skills can be impaired, which can result in a disruption of the parent-child bond [4, 6]. Moreover, parental mental illness is likely to be accompanied by other psychosocial stress factors, such as low socioeconomic status, unemployment, lack of close caregivers, and an increased risk of neglect and physical and sexual abuse [4, 8].

Growing up with a mentally ill parent is associated with adverse psychological and physical outcomes both in childhood [9] and adulthood [10, 11, 12]. The risk for children of parents with mental health problems to report mental health problems themselves is almost two times higher than the risk for children of parents without mental health problems [8]. The symptom severity of a parent’s mental illness is significantly associated with CAs’ psychiatric symptomatology [13, 14]. CA whose parents are both diagnosed with a mental illness are significantly more affected by psychiatric symptomatology than CA with only one mentally ill parent [13]. Moreover, parental psychopathology is significantly associated with lower health-related quality of life in CA [15]. Overall, CHIMPS have a greatly increased risk of developing a mental illness in the course of their lives [16, 17, 18]. According to a recent meta-analysis at the global level [19], the risk of developing the same mental illness as one’s parents ranges from 1% to 32%, depending on the parents’ diagnosis, whereas the risk of developing any mental illness ranges from 17% to 55%. In addition, CHIMPS make increased use of health and social services [20].

These adversities are influenced by both genetic and environmental factors, which requires a biopsychosocial perspective on developmental psychopathology [21, 22]. There is evidence that social support is associated with better mental health [23] and a better health-related quality of life [15] for CA. Furthermore, parents’ coping with their illness [24], overall family functioning [23], and parenting behavior–especially the mother-child relationship [22, 25]–are relevant to CAs’ mental health. These findings indicate that there are modifiable factors related to the mental health and health-related quality of life of CHIMPS.

In recent years, many family-oriented interventions for families with parents suffering from mental illness have been developed [26, 27]. Such interventions can reduce the risk of CA developing psychological symptoms by almost 50% [28], and specifically decreased internalizing symptoms [27, 28]. However, family-oriented services are still underrepresented, and there is a lack of high-quality evaluated interventions [26, 27]. Most evaluations are limited in their significance due to small sample sizes and short follow-up assessments [29]. A meta-analysis of randomized controlled trials (RCTs) on the prevention of depression in offspring of parents with depression revealed a significant effect at post-intervention assessments, but not across medium and long-term follow-up assessments [30]. Moreover, CA are not sufficiently considered when their mentally ill parents are admitted to and treated in psychiatric care [4]. The German health care system is predominantly individual-centered and lacking family-friendly structures in psychiatric clinics [31].

However, affected families need access to low-threshold, non-stigmatizing services that meet their needs [25, 32]. Improved care for CHIMPS is advisable from both a health economic and ethical perspective [33, 34]. To prevent pathologies of CA at risk, or to meet them therapeutically in time, it is necessary to examine the CAs’ mental health as early as possible. Demonstrating the effectiveness of a family intervention that includes a psychiatric screening of all family members and aims to improve CAs’ mental health and health-related quality of life is necessary to implement such interventions in routine care.

The CHIMPS program [35] was developed at the Center for Psychosocial Medicine at the University Medical Center Hamburg-Eppendorf to close this care gap. The objective of this trial was to evaluate the long-term effectiveness of the CHIMPS intervention [35] by comparing an intervention group (IG) to a control group (CG) that received Treatment-As-Usual (TAU). We assumed that the intervention would result in an improvement in the mental health and a higher health-related quality of life for CA compared to the CG in the long term. Specifically, the following hypotheses were tested:

### Hypothesis 1

CA (with and without mental health problems) in the IG will exhibit on average less mental health problems and a higher health-related quality of life compared to the CG 18 months after baseline assessment (population: all CA).

### Hypothesis 2

The proportion of CA with mental health problems in the IG will be lower compared to the CG 18 months after baseline assessment (population: CA with initial mental health problems, assessed by the German version of the Child Behavior Checklist/4–18 (CBCL/4–18 [36], hereinafter referred to as CBCL) cut-off T-score > 63).

### Hypothesis 3

CA with initial mental health problems in the IG will show a better health-related quality of life compared to the CG 18 months after baseline assessment (population: CA with initial mental health problems, assessed by the CBCL cut-off T-score > 63).

Our primary analysis was conducted using a sample of CA who had received a psychiatric diagnosis at baseline. We acknowledge that all CHIMPS constitute a high-risk population. However, our primary objective was to evaluate treatment effects among children with established psychopathology rather than preventive effects among at-risk children without a current disorder. We therefore predefined the primary analysis in a subgroup with a psychiatric diagnosis, for whom changes in psychiatric symptoms are most clinically meaningful and directly address the intervention’s therapeutic effectiveness.

### Hypothesis 4

(primary hypothesis): The proportion of CA with a psychiatric diagnosis in the IG will be lower compared to the CG 18 months after baseline assessment (population: CA with an initial psychiatric diagnosis, assessed by the German version of the Schedule for Affective Disorders and Schizophrenia for School-Age Children-Present and Lifetime Version (K-SADS-PL [37], parent interview).

### Hypothesis 5

CA with an initial psychiatric diagnosis in the IG will show a better health-related quality of life compared to the CG 18 months after baseline assessment (population: CA with an initial psychiatric diagnosis, assessed by the K-SADS-PL, parent interview).

## Methods

### Study design

CHIMPS was a prospective, two-arm, cluster-randomized, controlled, open, multicentric trial. Participants were examined at baseline (T1), as well as 6 (T2), 12 (T3), and 18 months (T4) after baseline. After participating family members had provided informed written consent, they completed the baseline assessment. After the baseline assessment, a family was randomized to either the IG or the CG. Computerized lists for family-wise (= cluster) randomization with an allocation ratio of 1:1 stratified by centers with a variable block length were used. These lists were prepared by the Institute of Medical Biometry and Epidemiology at the University Medical Center Hamburg-Eppendorf. The central allocation procedure was managed by the coordinating study center in Hamburg to guarantee allocation concealment. Outcomes were assessed from the mentally ill parents’, the second parents’ and the CAs’ (10 years and older) perspective. A blinded external rater interviewed one parent for each participating child, and in addition all CA aged 10 years and older. Interviews were audiotaped for quality control. The participants completed the questionnaires in a paper-and-pencil format at their home. Baseline interviews were carried out in person, follow-up interviews by telephone. After the baseline assessment and randomization, families were informed about their allocation to IG versus CG. For families in the IG, study therapists were informed and the intervention was initiated. Six, 12, and 18 months later, families were contacted by the study team for follow-up assessments.

### Participants

The participating families were recruited from in- and outpatient departments of psychiatric clinics at seven study centers – University Medical Center Hamburg-Eppendorf, Germany; University Medical Center Leipzig, Germany; Ulm University, Department of Psychiatry and Psychotherapy II, Ulm-Günzburg, Germany; Medical Center Vitos Clinic, Wiesbaden Rheingau, Germany; LWL Community Hospitals, Gütersloh-Paderborn, Germany; Charité – Universitätsmedizin Berlin, Germany; and Center of Social Pediatrics, Winterthur, Switzerland. Recruitment took place at both university and communal clinics. A study team member was in contact with psychiatric and/or psychological care providers. If a patient gave their consent to their care provider, they were contacted by the study team to receive information about the study. Families with at least one mentally ill parent and at least one child aged 3–19 years were eligible for the study. Confirmation of a parental psychiatric diagnosis was provided via a doctor’s note. Both couples and single parents could take part and participation was not limited to biological parents. Furthermore, regular contact between the mentally ill parent and their child(ren), sufficient knowledge of the German language, and written informed consent were required for participation. Parents or CA with severe psychiatric disorders and impairments with acute symptoms such as suicidal tendencies, self-injurious behavior, or acute psychotic symptoms were excluded from participation and referred to inpatient treatment. There were no withdrawals in this study, only lost to follow-up. A family was defined as lost to follow-up if the whole family did not provide further data. Families received financial compensation of 50€ for their participation.

### CHIMPS intervention

The psychodynamic and transdiagnostic CHIMPS intervention [35] is designed for families with at least one parent who has a mental illness and at least one child between the ages of 3 and 21. The concept of family is defined broadly here: Both single parents and close caregivers who are not biologically related to the child may participate in the intervention. The intervention is not suitable for individuals with acute severe psychiatric conditions that require inpatient treatment.

CHIMPS [35] is based on the following pillars: Firstly, a *Model of psychosocial development conditions for children of mentally ill parents* [35, 38], identifying three key factors for CAs’ development. (a) Type and adequacy of coping with the parental mental illness; (b) scope and quality of interpersonal relationships; and (c) family dynamics. Secondly, psychoanalytic family therapy [39]. Thirdly, evidence for acceptance of family-based interventions among families with a mentally ill parent [35]. Finally, the setting and number of sessions are based on an internationally evaluated intervention by Beardslee [40].

The intervention takes parent-level, dyad-level, and family-level factors into account, as processes on all these levels contribute to the development and maintenance of CAs’ psychopathology [41]. It comprises approximately eight sessions (60–90 min each) – three only with parents, one per child only with children, and three with the whole family, which typically span half a year. If family members have additional needs, there may be an extra session with the parents and/or for each child and/or with the whole family, which is decided individually for each family. Families with separated parents may require more parent sessions. This can happen if parents decide later to attend sessions together or choose to attend separate sessions. The total number of children sessions depends (also) on the number of children in a family. The number of family sessions may increase if the parent with a mental illness is hospitalized and the other family members continue attending counseling during the hospitalization.

The intervention aims to bring the unconscious conflicts among individual family members, family subsystems, or the family as a whole into consciousness. To this end, psychodynamic intervention techniques (clarifying, confronting, and interpreting) are used. Conscious conflicts can then be addressed in the therapeutic process. The psychodynamic focus of the intervention is evident in the therapists’ psychoanalytic approach and their psychoanalytic understanding of a family’s issues. However, general psychotherapeutic elements, such as psychoeducation, and elements based on behavioral therapy, such as positive reinforcement, are integrated. The therapists primarily address issues such as individual, couple-based, and intrafamilial coping with the parental mental illness, relationships within and outside the family, and couple and family dynamics.

During the parent sessions, therapists assess the family’s current situation, including family structure, socioeconomic circumstances, and current stressors. They also explore the parental mental illness (e.g., symptom severity, illness course, and treatment history), parents’ understanding and communication about the illness, family relationships, available social support, and concerns about their children. In addition, children’s needs, coping, caregiving arrangements during acute phases of illness, and their strengths and challenges are discussed. Finally, parents identify goals at the individual, couple, and family levels.

During the children sessions, therapists explore the children’s current situation at home and in kindergarten, school, or work. Discussions focus on their understanding of the parental illness, related thoughts, feelings, concerns, communication about the illness, coping strategies, use of support services, family and peer relationships, and illness-related changes in family roles. In addition, children reflect on their strengths, challenges, resources, and personal goals.

If the necessary resources are available, diagnostic interviews with all family members can be conducted in parallel with the children’s sessions to identify current mental health impairments or disorders.

Family sessions provide a space to explore family dynamics and promote open communication. They address topics and perspectives that emerged during the individual sessions, with a focus on coping, relationships, family dynamics, and current conflicts. Therapists support discussions about the parent’s illness, help reduce children’s feelings of guilt, and adapt the process to the family’s needs and pace. In the final family session, therapy is concluded, additional support services are discussed, and families are encouraged to seek further help if needed.

The main goals of CHIMPS are to provide psychoeducation about the parental illness, improve family communication, strengthen coping and relationships within and outside the family, and reduce social isolation. The intervention also aims to increase parents’ understanding of risk and protective factors for child development, provide information about available support services, and encourage families to use these resources.

All therapists received a comprehensive 2-day training based on the CHIMPS manual [35]. To monitor manual adherence, therapists completed a self-report checklist documenting the main topics covered after each session. The checklist included the core content areas specified in the CHIMPS manual [35]. In addition, therapists received ongoing supervision, both in person and online, from licensed therapists of the study team. If participants agreed, all sessions were video-recorded for quality control. The intervention was applied between T1 and T2.

TAU could comprise, for instance, individual psychotherapy for CA and/or parents, psychiatric treatment for CA and/or parents, and support at youth welfare services. Families in the CG were free to use these support services. However, no treatment was offered to families in the CG as part of the project. Families requiring additional support were referred to appropriate services. Families in the IG were also allowed to receive additional treatment.

### Materials

Sociodemographic information including age, sex, number of children, educational and employment status, somatic and psychiatric illnesses, history of treatment, and current treatment were collected from all participating family members at baseline using standardized questionnaires. Parents provided information about themselves and their children, while CA aged 10 years and older provided information about themselves. Thus, for children aged 10 and older, parent-rated and self-rated assessments were provided.

#### Schedule for affective disorders and schizophrenia for school-age children-present and lifetime version

The primary outcome was the CAs’ psychopathology measured by the K-SADS-PL. The K-SADS-PL is a semi-structured diagnostic interview developed for the assessment of current and past episodes of mental illness in CA according to DSM-III-R and DSM-IV criteria. The result of the K-SADS-PL comprises three categories — either *Confirmed diagnosis*, *Near-diagnosis*, or *No diagnosis*. The interview takes approximately 45–75 min to complete [42]. The K-SADS-PL has been widely used in research due to the good reliability and validity of its original version [43]. For the primary analysis in this study, CA were classified as having a psychiatric diagnosis if at least one interview with parent or CA indicated a *Confirmed diagnosis*. The type and number of diagnoses were recorded but did not influence this classification. Student assistants under supervision administered the K-SADS-PL to family members.

#### Child behavior checklist/youth self report

As a secondary outcome, the CAs’ psychopathology was further assessed with the CBCL and the Youth Self Report (YSR [36]). In this study, only the second part of the CBCL was administered, a parent-rated 120-item assessment of behavioral problems, emotional problems, and physical complaints in the past six months [36]. Parents are asked to rate the occurrence of a behavior on a 3-point Likert scale (0 = *Not true*, 1 = *Somewhat or sometimes true*, 2 = *Very true or often true*). The questionnaire takes approximately 15–20 min to complete. Due to its satisfactory psychometric properties, the CBCL is a widely used questionnaire in research [44, 45].

In this study, only the second part of the YSR was administered, a self-rated 119-item assessment of behavioral problems, emotional problems, physical complaints, and socially desirable behaviors in the past six months [36]. The scoring system and processing time correspond to those of the CBCL. Due to its satisfactory psychometric properties, the YSR is a widely used questionnaire in research [36, 46]. In this study, CA aged 10 years and older completed the YSR. CA were classified as having mental health problems if the CBCL assessed by a parent or the YSR assessed by CA or both resulted in a T-score > 63.

#### KIDSCREEN-27/KIDSCREEN-10

As a secondary outcome, the CAs’ health-related quality of life was assessed with the German version of the KIDSCREEN-27 — specifically, its global score, the KIDSCREEN-10 [47]. The KIDSCREEN-27 is a short version of the KIDSCREEN-52. Both a self-rated version (self) and a parent-rated version (proxy) are available. In this study, both parents and CA aged 10 years and older completed the KIDSCREEN-27. Health-related quality of life is assessed on a 5-point Likert scale (1 = *excellent*, 2 = *very good*, 3 = *good*, 4 = *fair*, 5 = *poor* [reversed]; or 1 = *not at all*, 2 = *slightly*, 3 = *moderately*, 4 = *very*, 5 = *extremely*; or 1 = *never*, 2 = *seldom*, 3 = *quite often*, 4 = *very often*, 5 = *always*) for the past week. The questionnaire takes approximately 10–15 min to complete. The KIDSCREEN-27 has demonstrably good psychometric properties [47, 48]. The KIDSCREEN-10 is a global value for health-related quality of life and comprises ten items of the KIDSCREEN-27. Given that it provides a representative summary score of the KIDSCREEN-27, it was used in the present analyses.

### Analysis populations

The primary analysis population was the Intention-To-Treat (ITT) population. The ITT population consisted of all families randomized, with at least one valid follow-up value of the outcome under consideration (called ITT 1 population). For hypothesis 1, analyses were run with the ITT1 population. For hypotheses 2 and 3, we limited the ITT1 population to all CA with initial mental health problems (CBCL T-score > 63) and at least one valid follow-up value for the outcome under consideration (called ITT2 population). For hypotheses 4 and 5, we limited the ITT1 population to all CA with an initial psychiatric diagnosis according to the K-SADS-PL (parent interview) and at least one valid follow-up value for the outcome under consideration (called ITT3 population). All analyses were conducted using parent-rated assessments. Hypothesis 1, 3, and 5 were additionally analyzed with data from the CAs’ perspective.

In the Per-Protocol (PP) populations, families with major protocol deviations were excluded, resulting in three corresponding PP populations. If a family completed fewer than five therapy sessions, it was considered a major protocol deviation. No protocol deviations were defined in the CG.

### Data analysis

A detailed description of all statistical procedures, including calculation of the sample size, can be found in the corresponding study protocol [49].

Baseline characteristics are presented with absolute and relative frequencies for categorical data, and with mean, standard deviation, median, minimum, and maximum for continuous data. The primary endpoint was analyzed using a mixed logistic regression. A random effect for family and for child nested within family was included. Additionally, the following fixed effects were included: recruitment center, treatment group, time, the interaction between treatment group and time, and variables which were fairly (using descriptive *p*-values < 0.05) unbalanced between treatment groups at baseline. For this analysis, we combined the categories *Near-diagnosis* and *No diagnosis* into one category. The result of the primary endpoint analysis was the treatment group contrast (CHIMPS intervention versus TAU) at T4. For *p*-values of the interaction between treatment group and time > 0.05, an effect averaged over time was reported. The primary endpoint analysis was based on the ITT3 population.

To assess which baseline variables were unbalanced between treatment groups, we created a model dependent on the scale level of the baseline variable under consideration. For continuous variables, we used a linear (mixed) model; for binary variables, (mixed) logistic regression; for ordinal variables, (mixed) ordinal regression; and for nominal variables, (mixed) nominal regression. In each model, family was included as a random effect, and recruitment center and treatment group as fixed effects. When assessments on CA or from the CAs’ perspective were evaluated, mixed models with family as a random effect were used. When assessments on parents were evaluated, this random effect was not included in the model. The baseline variables under consideration were the variables listed in Supplementary Material 1.a and the primary and secondary endpoints assessed at baseline (Supplementary Material 1.b). The following baseline variables were found to be unbalanced: CBCL *Delinquent Behavior* score, the mentally ill parent’s *Task Fulfillment* score (from the questionnaire Allgemeiner-Familienbogen, FB-A [50], assessing family relationships), and the mentally ill parent’s *Trivialization and Wishful Thinking* score (from the questionnaire Freiburger Fragebogen zur Krankheitsverarbeitung, FKV [51], assessing coping).

To assess the robustness of the results, the primary endpoint analysis was repeated using imputed baseline values and two different imputation methods for missing follow-up data (see Supplementary Material 1.c for further details).

The primary endpoint analysis was repeated in the PP3 population, which was limited to CA of the ITT3 population whose family had participated in at least five therapy sessions. As a further sensitivity analysis, we repeated the primary endpoint analysis with a second categorization of having a psychiatric diagnosis according to K-SADS-PL (*Near diagnosis*/*Confirmed diagnosis* versus *No diagnosis*).

Pre-specified subgroup analyses of the primary endpoint were conducted with the variables recruitment center, type of clinic (university versus communal clinic), CAs’ age (3 to 13 years versus 14 to 19 years), and CAs’ sex (male versus female). In the subgroup analyses, a separate model was carried out for each of the variables. The model of the primary endpoint analysis was successively supplemented by one of the variables and its interaction with the treatment group variable.

The secondary endpoint *mental health problems according to CBCL (yes/no)* was analyzed with a mixed logistic regression analogous to the primary endpoint analysis. In addition, the CBCL score was treated as a continuous variable. All other secondary endpoints were continuous as well. For each secondary endpoint, the change from baseline was used as the dependent variable in a linear mixed model with the same fixed and random effects as specified for the primary endpoint analysis. The baseline value of the respective endpoint was additionally included as a covariate.

R version 4.2.3 [52] was used for analysis.

## Results

### Participants

**Fig. 1:**
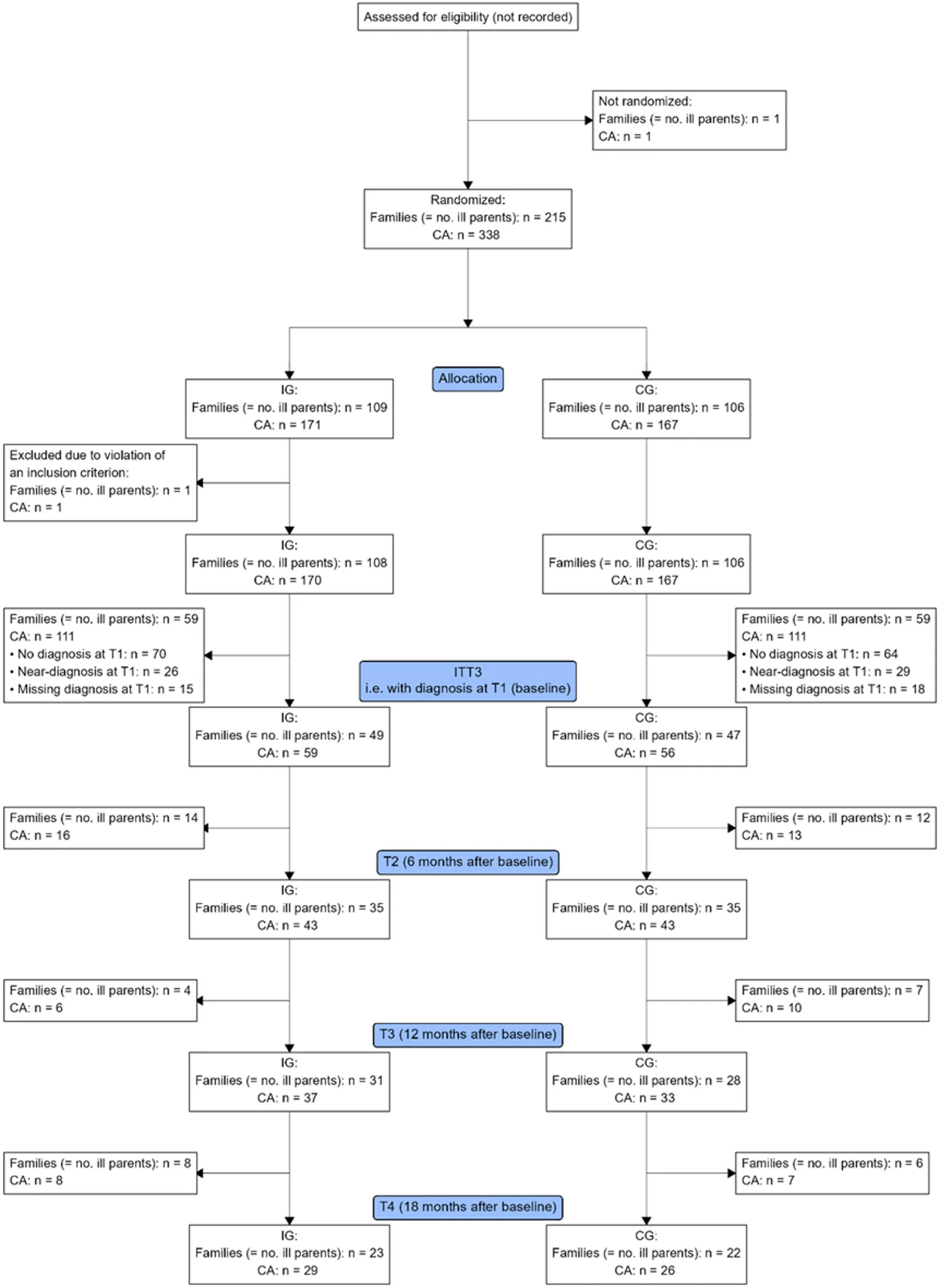
CONSORT flow diagram depicting the number of families and CA who were randomized, received versus did not receive the allocated intervention, were lost to follow-up, and included in versus excluded from the primary endpoint analysis. CA = children and adolescents; CG = Control Group; IG = Intervention Group; ITT = Intention-To-Treat; no. = number

A CONSORT flow diagram (Fig. 1) is used to summarize the number of families (= number of mentally ill parents) and the number of CA who were randomized, received versus did not receive the allocated intervention, were lost to follow-up, and included in versus excluded from the primary endpoint analysis.

Two hundred fifteen families were randomized, of which 109 were allocated to the IG and 106 to the CG. One family of the IG was excluded due to violation of an inclusion criterion. Sixty-two of these families were recruited in Hamburg, 38 in Leipzig, 49 in Ulm-Günzburg, twelve in Wiesbaden Rheingau, 34 in Gütersloh-Paderborn, 17 in Berlin, and three in Winterthur.

Sociodemographic information on the 214 mentally ill parents including age, sex, number of children, marital status, education, and professional qualification at baseline is presented in Table 1. Information on physical and mental illness, history of treatment, and current treatment, the frequency of specific ICD-10 diagnostic classes, and the frequency of different quantities of diagnoses per person at baseline are presented in Supplementary Material 2.a, 2.b, and 2.c.

**Table 1:** Sociodemographic information on mentally ill parents

	Total (*n* = 214)	IG (*n* = 108)	CG (*n* = 106)
Age			
Information missing	4/214 (1.9%)	1/108 (0.9%)	3/106 (2.8%)
*M* (*SD*)	40.1 (7.1)	39.9 (7.6)	40.4 (6.6)
Median (Min-Max)	40.0 (23.0–57.0)	40.0 (23.0–55.0)	40.0 (24.0–57.0)
Sex			
Information missing	5/214 (2.3%)	3/108 (2.8%)	2/106 (1.9%)
Female	157/209 (75.1%)	80/105 (76.2%)	77/104 (74.0%)
Male	52/209 (24.9%)	25/105 (23.8%)	27/104 (26.0%)
Number of children			
Information missing	6/214 (2.3%)	4/108 (3.7%)	2/106 (1.9%)
1	79/208 (38.0%)	41/104 (39.4%)	38/104 (36.5%)
2	86/208 (41.3%)	41/104 (39.4%)	45/104 (43.3%)
3	29/208 (13.9%)	15/104 (14.4%)	14/104 (13.5%)
4	11/208 (5.3%)	6/104 (5.8%)	5/104 (4.8%)
5	3/208 (1.4%)	1/104 (1.0%)	2/104 (1.9%)
Number of children aged 4–18 years			
Information missing	11/214 (5.1%)	8/108 (7.4%)	3/106 (2.8%)
0	3/203 (1.5%)	1/100 (1.0%)	2/103 (1.9%)
1	110/203 (54.2%)	56/100 (56.0%)	54/103 (52.4%)
2	69/203 (34.0%)	33/100 (33.0%)	36/103 (35.0%)
3	16/203 (7.9%)	7/100 (7.0%)	9/103 (8.7%)
4	4/203 (2.0%)	3/100 (3.0%)	1/103 (1.0%)
5	1/203 (0.5%)	0/100 (0.0%)	1/103 (1.0%)
Marital status			
Information missing	8/214 (3.7%)	4/108 (3.7%)	4/106 (3.8%)
Single	53/206 (25.7%)	32/104 (30.8%)	21/102 (20.6%)
Married	112/206 (54.4%)	53/104 (51.0%)	59/102 (57.8%)
Divorced	36/206 (17.5%)	17/104 (16.3%)	19/102 (18.6%)
Widowed	5/206 (2.4%)	2/104 (1.9%)	3/102 (2.9%)
Highest level of education			
Information missing	13/214 (6.1%)	7/108 (6.5%)	6/106 (5.7%)
Secondary school (nine years of schooling)	42/201 (20.9%)	23/101 (22.8%)	19/100 (19.0%)
Secondary school (ten years of schooling)	91/201 (45.3%)	51/101 (50.5%)	40/100 (40.0%)
High school diploma	64/201 (31.8%)	25/101 (24.8%)	39/100 (39.0%)
Without school leaving certificate	4/201 (2.0%)	2/101 (2.0%)	2/100 (2.0%)
Highest professional qualification			
Information missing	28/214 (13.1%)	14/108 (13.0%)	14/106 (13.2%)
Apprenticeship	96/186 (51.6%)	55/94 (58.5%)	41/92 (44.6%)
Master craftsman/…….Technical college	27/186 (14.5%)	15/94 (16.0%)	12/92 (13.0%)
University	40/186 (21.5%)	14/94 (14.9%)	26/92 (28.3%)
Without professional qualification	22/186 (11.8%)	10/94 (10.6%)	12/92 (13.0%)
Other	1/186 (0.5%)	0/94 (0.0%)	1/92 (1.1%)

CG = Control Group; IG = Intervention Group; *M* = Mean; Max = Maximum; Min = Minimum; *SD* = Standard Deviation

Three hundred thirty-eight CA were randomized, of which 171 were allocated to the IG and 167 to the CG. Data of one child or adolescent of the IG were excluded due to their family’s violation of an inclusion criterion. Sociodemographic information on the 337 CA including age, sex, and current treatment at baseline — as reported by their parents — is presented in Table 2. The frequency of specific ICD-10 diagnostic classes and the frequency of different quantities of diagnoses per child/adolescent at baseline are presented in Supplementary Material 2.d and 2.e.

**Table 2:** Sociodemographic information on and medical status of CA

	Total (*n* = 337)	IG (*n* = 170)	CG (*n* = 167)
Age			
Information missing	4/337 (1.2%)	2/170 (1.2%)	2/167 (1.2%)
*M* (*SD*)	9.0 (1.0–19.0)	9.0 (1.0–19.0)	9.0 (1.0–19.0)
Median (Min-Max)	40.0 (23.0–57.0)	40.0 (23.0–55.0)	40.0 (24.0–57.0)
Sex			
Information missing	2/337 (0.6%)	1/170 (0.6%)	1/167 (0.6%)
Female	175/335 (52.2%)	89/169 (52.7%)	86/166 (51.8%)
Male	160/335 (47.8%)	80/169 (47.3%)	80/166 (48.2%)
Current psychiatric/psychotherapeutic outpatient treatment			
Information missing	17/337 (5.0%)	12/170 (7.1%)	5/167 (3.0%)
No	274/320 (85.6%)	140/158 (88.6%)	134/162 (82.7%)
Yes	46/320 (14.4%)	18/158 (11.4%)	28/162 (17.3%)
Frequency of outpatient treatment			
Information missing	294/337 (87.2%)	153/170 (90.0%)	141/167 (84.4%)
Several times a week	2/43 (4.7%)	0/17 (0.0%)	2/26 (7.7%)
Once a week	13/43 (30.2%)	7/17 (41.2%)	6/26 (23.1%)
Biweekly	8/43 (18.6%)	3/17 (17.6%)	5/26 (19.2%)
Once a month or less	20/43 (46.5%)	7/17 (41.2%)	13/26 (50.0%)

CA = children and adolescents; CG = Control Group; IG = Intervention Group; *M* = Mean; Max = Maximum; Min = Minimum; *SD* = Standard Deviation

For 38.5% of the families in the IG, no therapy sessions were recorded; for 16.5% of the families, one to three; for 27.6% of the families, four to six; for 13.7% of the families, seven to nine; and for 3.7%, ten or eleven. For 34% of the families, a preliminary session with the family was documented.

### Primary endpoint

**Fig. 2:**
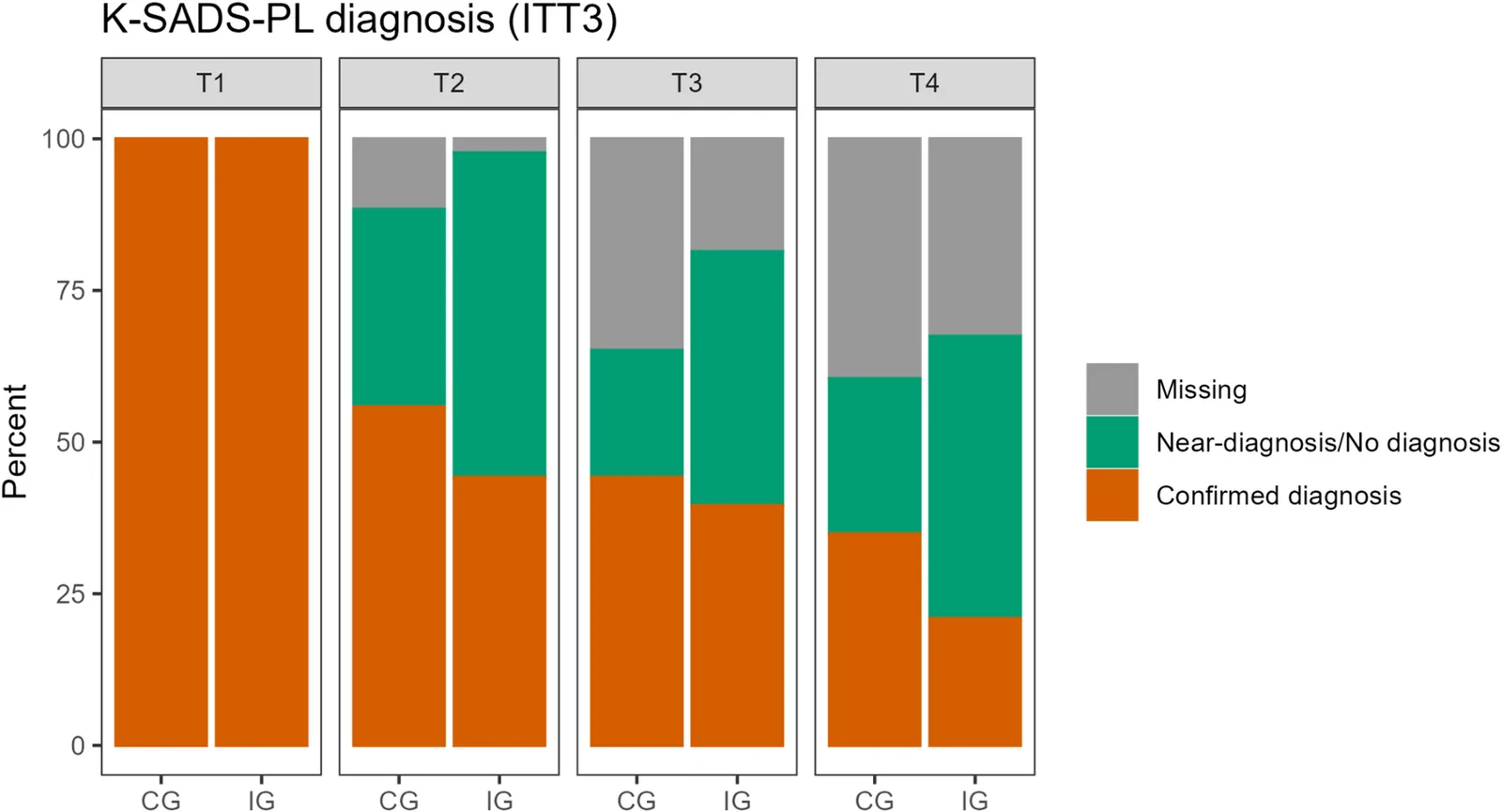
K-SADS-PL diagnoses in IG and CG in the ITT3 population over time. CG = Control Group; IG = Intervention Group; ITT = Intention-To-Treat; K-SADS-PL = Schedule for Affective Disorders and Schizophrenia for School-Age Children-Present and Lifetime Version

At baseline, 86 CA had a psychiatric diagnosis according to K-SADS-PL and a valid follow-up value. At T4, 43.6% of CA who had received a psychiatric diagnosis as baseline still had a *Confirmed diagnosis*, while 56.4% had a *Near-diagnosis* or *No diagnosis*. Figure 2 displays the proportion of CA (IG and CG separately) in the ITT3 population who had a missing value, a *Near-diagnosis*/*No diagnosis*, and a *Confirmed diagnosis* according to the K-SADS-PL, separately for all four measurement points.

The results of the primary endpoint analysis are presented in Table 3. The interaction *p*-value between treatment group and time was 0.836, hence the treatment effect does not need to be differentiated between measurement points. The treatment effect (odds ratio [OR]) for all measurement points was 0.52 (95% CI: 0.25; 1.09), which means that less events (*Confirmed diagnosis* according to K-SADS-PL) were observed in the IG compared to the CG, yet without reaching statistical significance.

**Table 3:** Results of primary endpoint analysis on K-SADS-PL diagnoses in the ITT3 population

Outcome	IG	CG	IG versus CG			
	Events/total (%)	Events/total (%)	Adj. OR (95% CI)	*p*-value	*p*-value interaction (treatment group-time)	*n*
K-SADS-PL diagnosis			0.52 (0.25; 1.09)	0.0844	0.8356	189
T2	19/42 (45.24)	24/38 (63.16)				
T3	17/35 (48.57)	19/28 (67.86)				
T4	9/29 (31.03)	15/26 (57.69)				

Adj. OR = adjusted Odds Ratio; CG = Control Group; CI = Confidence Interval; IG = Intervention Group; ITT = Intention-To-Treat; K-SADS-PL = Schedule for Affective Disorders and Schizophrenia for School-Age Children-Present and Lifetime Version

### Secondary endpoints

#### Child behavior checklist

**Fig. 3:**
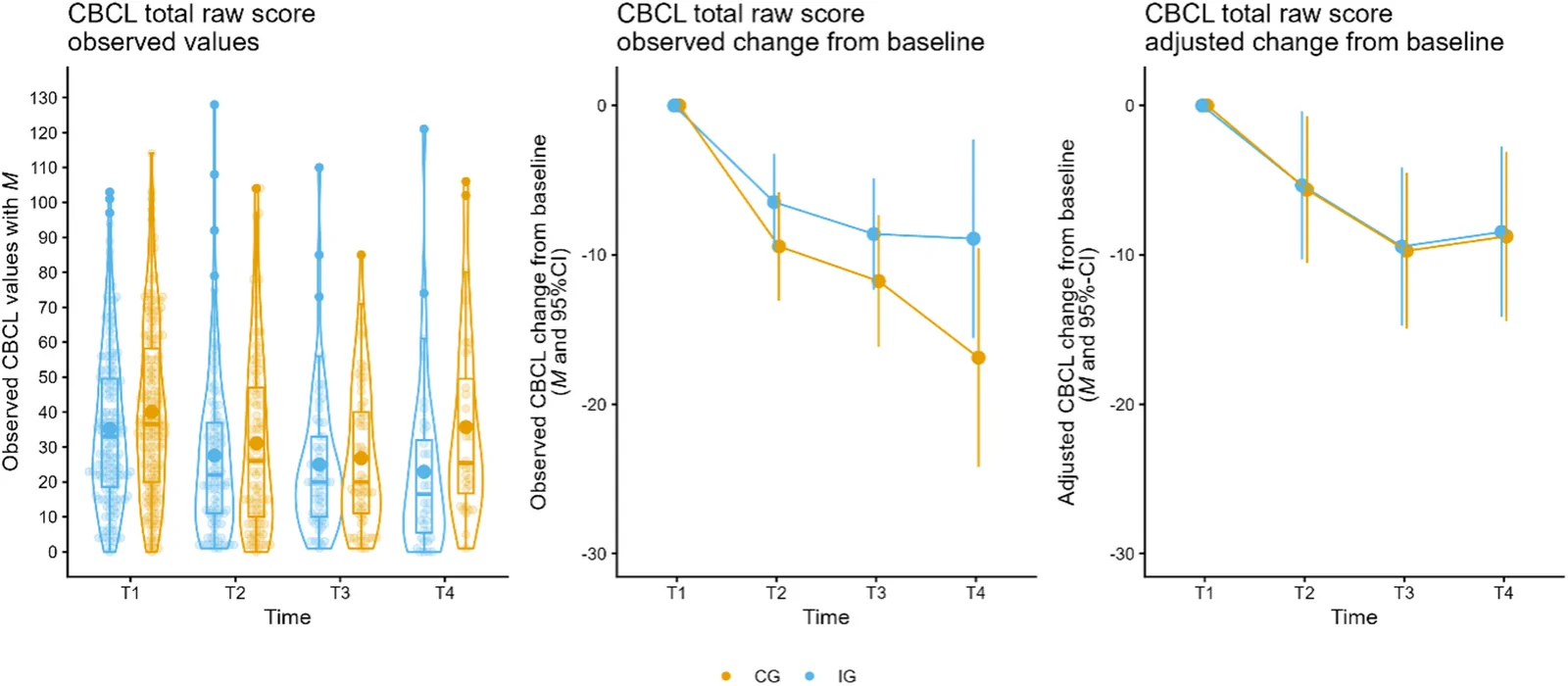
CBCL total raw score in IG and CG in the ITT1 population over time. Please note: The inconsistency between the left and middle panel at T4 is due to small sample size at T4 and a high proportion of CA with missing data at T1. CA = children and adolescents; CBCL = Child Behavior Checklist; CG = Control Group; IG = Intervention Group; ITT = Intention-To-Treat

At baseline, the mean CBCL total raw score was 37.7 (*SD* = 23.9), ranging from 0.0 to 114.0. At T4, the mean CBCL total raw score was 28.9 (*SD* = 26.4), ranging from 0.0 to 121.0. The results of the analysis of the continuous CBCL total raw score in the ITT1 population are illustrated in Fig. 3 and depicted in Table 4. The adjusted mean difference for all measurement points between the IG and the CG was 0.30 (95% CI: -4.44; 5.03), showing nearly no difference between treatment groups. The effect does not seem to differ over time, as depicted by the interaction *p*-value 0.992.

**Table 4:** Results of secondary endpoint analysis on CBCL total raw score in the ITT1 population

Outcome	IG			CG			IG versus CG			
	*n*	Obs. M (SD)	Adj. M (95% CI)	*n*	Obs. M (SD)	Adj. M (95% CI)	Adj. M diff.(95% CI)	*p*-value	*p*-value interaction (treatment group-time)	*n*
CBCL							0.30 (-4.44; 5.03)	0.9017	0.9922	367
T1	159	35.25 (22.63)		162	40.04 (24.89)					
T2-T1	91	-6.46 (15.74)	-5.34 (-10.29; -0.38)	93	-9.43 (17.94)	-5.64 (-10.56; -0.71)				
T3-T1	57	-8.60 (14.42)	-9.43 (-14.72; -4.15)	61	-11.75 (17.56)	-9.73 (-14.94; -4.52)				
T4-T1	34	-8.91 (19.79)	-8.46 (-14.16; -2.75)	32	-16.88 (21.14)	-8.76 (-14.42; -3.10)				

Adj. *M* = adjusted Mean; CBCL = Child Behavior Checklist; CG = Control Group; CI = Confidence Interval; diff. = difference; IG = Intervention Group; ITT = Intention-To-Treat; obs. *M* = observed Mean; *SD* = Standard Deviation

**Fig. 4:**
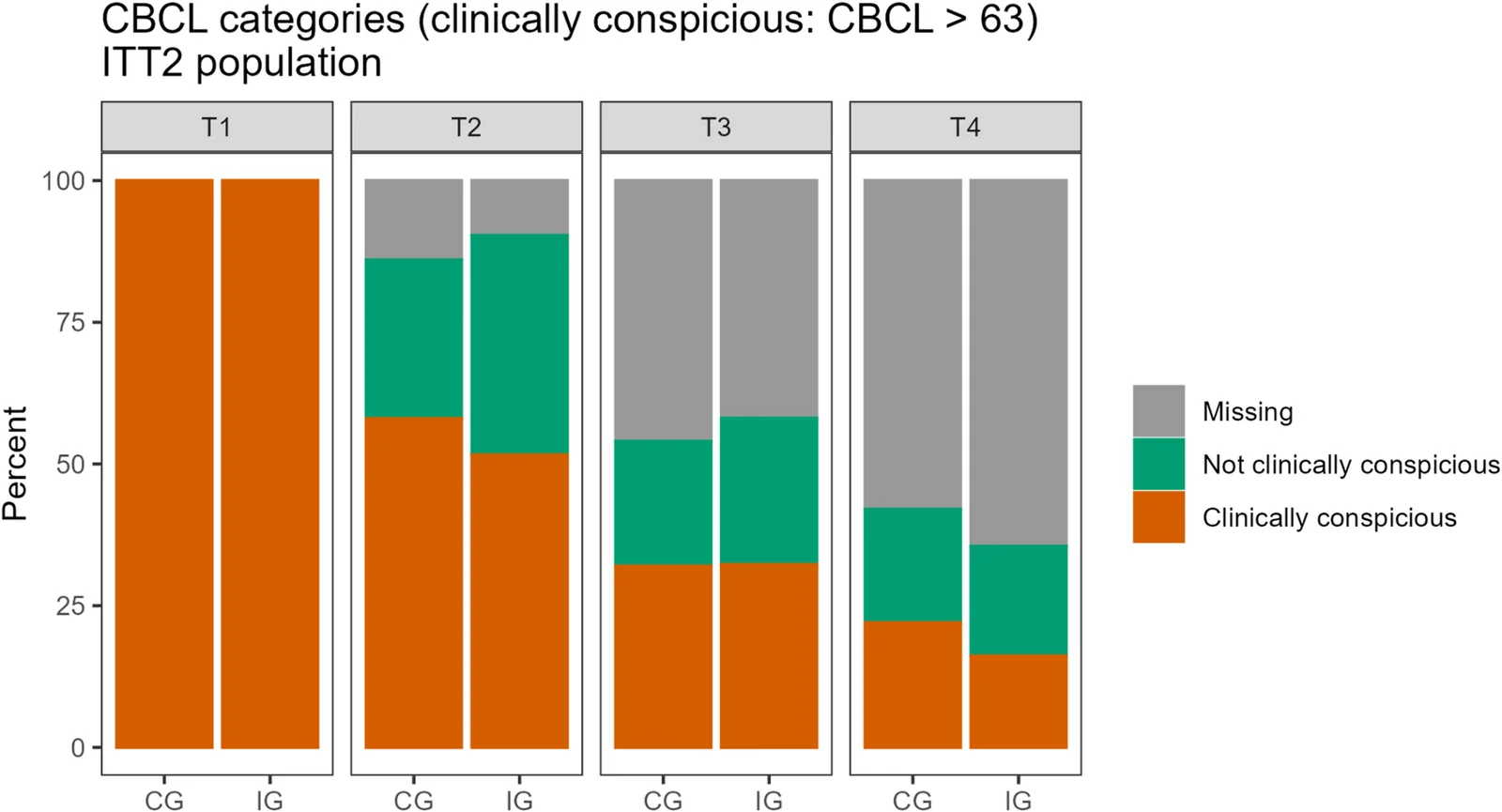
CBCL categories in IG and CG in the ITT2 population over time. CBCL = Child Behavior Checklist; CG = Control Group; IG = Intervention Group, ITT = Intention-To-Treat

Figure 4; Table 5 display the results of the analysis of the CBCL as binary endpoint in the ITT2 population. An OR of 0.79 (95%CI: 0.18; 3.47) was observed over all measurement points, hence less events (clinically conspicuous, defined by CBCL T-score > 63) were observed in the IG than in the CG. The effect does not seem to differ over time, as depicted by the interaction *p*-value 0.876.

**Table 5:** Results of secondary endpoint analysis on CBCL as binary endpoint in the ITT2 population

Outcome	IG	CG	IG versus CG			
	events/total (%)	events/total (%)	adj. OR (95% CI)	*p*-value	*p*-value interaction (treatment group-time)	*n*
CBCL (T > 63)			0.79 (0.18; 3.47)	0.7568	0.8761	147
T2	16/28 (57.14)	29/43 (67.44)				
T3	10/18 (55.56)	16/27 (59.26)				
T4	5/11 (45.45)	11/21 (52.38)				

Adj. OR = adjusted Odds Ratio; CBCL = Child Behavior Checklist; CG = Control Group; CI = Confidence Interval; IG = Intervention Group; ITT = Intention-To-Treat

#### KIDSCREEN-10 (proxy)

**Fig. 5:**
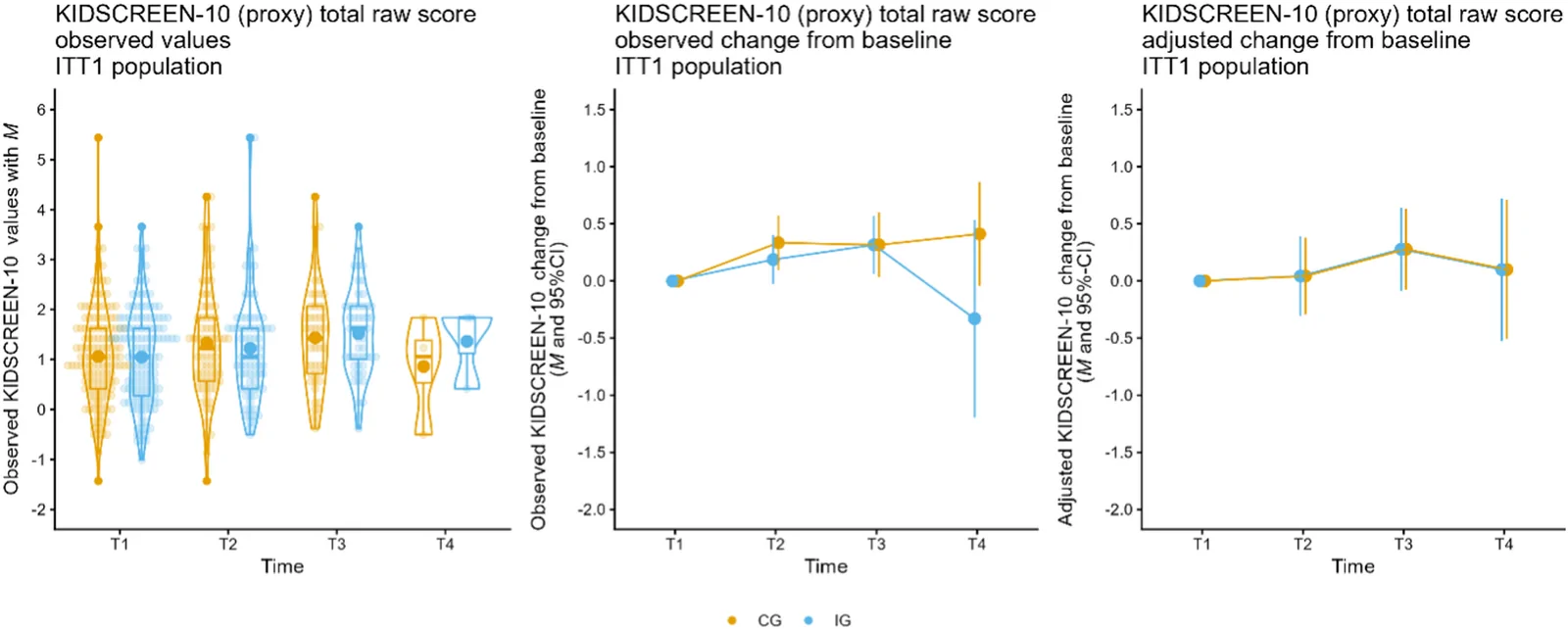
KIDSCREEN-10 (proxy) total raw score in IG and CG in the ITT1 population over time. Please note: The inconsistency between the left and middle panel at T4 is due to small sample size at T4 and a high proportion of CA with missing data at T1. CA = children and adolescents; CG = Control Group; IG = Intervention Group; ITT = Intention-To-Treat

At baseline, the mean KIDSCREEN-10 (proxy) total raw score was 1.05 (*SD* = 0.07), ranging from − 1,43 to 5.44. At T4, the mean KIDSCREEN-10 (proxy) total raw score was 1.08 (*SD* = 0.89), ranging from − 0.50 to 1.84. Figure 5 illustrates the results of the analysis of the KIDSCREEN-10 (proxy) total raw score in the ITT1 population, which are summed up in Table 6.

**Table 6:** Results of secondary endpoint analysis on KIDSCREEN-10 (proxy) total raw score in the ITT1 population

Outcome	IG			CG			IG versus CG			
	*n*	Obs. M (SD)	Adj. M (95% CI)	*n*	Obs. M (SD)	adj. M (95% CI)	M diff. (95% CI)	*p*-value	*p*-value interaction (treatment group-time)	*n*
KIDSCREEN-10							-0.00 (-0.30; 0.30)	0.9979	0.4718	232
T1	138	1.05 (0.95)		141	1.06 (0.98)					
T2-T1	66	0.19 (0.89)	0.04 (-0.30; 0.39)	71	0.33 (1.03)	0.04 (-0.29; 0.38)				
T3-T1	44	0.32 (0.86)	0.28 (-0.09; 0.64)	45	0.32 (0.97)	0.28 (-0.08; 0.63)				
T4-T1	3	-0.33 (0.77)	0.10 (-0.52; 0.72)	4	0.41 (0.46)	0.10 (-0.51; 0.71)				

Adj. *M* = adjusted Mean; CG = Control Group; CI = Confidence Interval; diff. = difference; IG = Intervention Group; ITT = Intention-To-Treat; obs. *M* = observed Mean; *SD* = Standard Deviation

Results of the analysis of the KIDSCREEN-10 (proxy) total raw score in the ITT2 population and ITT3 population can be found in Supplementary Material 2.f, 2.g, 2.h, and 2.i.

In all populations, (nearly) no treatment effect was observed (adjusted mean difference ≈ 0).

#### Youth Self Report

Self-assessments from 153 CA aged 10 years and older (72 IG, 81 CG) were available. At baseline, the mean YSR total raw score was 66.8 (*SD* = 20.2), ranging from 27.0 to 116.0. At T4, the mean YSR total raw score was 54.3 (*SD* = 17.7), ranging from 23.0 to 93.0. Results of the analysis of the YSR total raw score in the ITT1 population can be found in Supplementary Material 2.j. and 2.k. The adjusted mean difference for all measurement points between IG and CG was − 6.51 (-13.90; 0.88). The effect does not seem to differ over time, as depicted by the interaction *p*-value 0.354.

#### KIDSCREEN-10 (self)

At baseline, the mean KIDSCREEN-10 (self) total raw score was 0.94 (*SD* = 1.04), ranging from − 1.21 to 4.70. At T4, the mean KIDSCREEN-10 (self) total raw score was 1.24 (*SD* = 0.98), ranging from − 0.56 to 2.95.

Results of the analysis of the KIDSCREEN-10 (self) total raw score in the ITT1 population, ITT2 population, and ITT3 population can be found in Supplementary Material 2.l, 2.m, 2.n, 2.o, 2.p, and 2.q.

### Sensitivity analyses

Twenty-three families (29 CA) deviated from the protocol. The analysis based on the PP3 population and imputed data supported the primary endpoint analysis (see Supplementary Material 2.r and 2.s). A sensitivity analysis of the primary endpoint analysis, combining the categories *Confirmed diagnosis* and *Near-diagnosis* of the K-SADS-PL into one category, resulted in an OR of 0.72 (95% CI: 0.30; 1.68). The results can be seen in Supplementary Material 2.t.

### Subgroup analyses

Neither recruitment center (interaction *p*-value: 0.196), nor type of clinic (university clinic versus communal clinic, interaction *p*-value: 0.855), nor CAs’ age (3–13 years versus 14–19 years, interaction *p*-value: 0.837), nor CAs‘ sex (male versus female, interaction *p*-value: 0.861) yielded a relevant interaction with the treatment group (see Supplementary Material 2.u), hence it seems not necessary to differentiate the treatment effect for these subgroups.

## Discussion

The CHIMPS study conducted a high-quality evaluation of an intervention for CHIMPS and their families. The primary aim was to analyze whether the intervention would reduce the proportion of CA with a psychiatric diagnosis more than TAU over a period of 18 months. Secondary analyses examined long-term improvements in mental health symptoms following the intervention both in the entire group of CA and in a group of CA with initial mental health problems. We further analyzed long-term improvements in CAs’ health-related quality of life in all three populations.

The outcomes of interest — psychopathology and health-related quality of life — were assessed at several measurement points from different perspectives, resulting in a more comprehensive representation of the variables [28, 53].

A network of participating study centers was successfully established and awareness for the risk group CHIMPS was raised [49]. Overall, analyses revealed that the group differences were small and not consistent with regard to an advantage or disadvantage of the IG. The primary hypothesis test, based on the ITT3 population, showed no statistical significance. The sensitivity analyses supported the primary endpoint analysis. None of the subgroup analyses indicated a relevant difference between subgroup categories. In total, both the IG and the CG improved in most outcomes over time, with the following exceptions: CA of both groups in the ITT3 population deteriorated over time in parent-rated health-related quality of life. Also, CA of the CG in both the ITT1 and ITT2 population rated their health-related quality of life to worsen over time. The meaningfulness of results is limited due to the small sample size.

Our findings contrast with meta-analyses that underlined the effectiveness of interventions in improving the mental health of CHIMPS [27, 28]. However, Moltrecht et al. [27] pointed out the low quality of some of the studies, which may have exaggerated the results. Lannes et al. [28] underlined that only few trials were included in their meta-analysis, and these trials often reported only small samples. Effects tended to be small [27, 28]. These limitations point to the importance of further high-quality evaluations of interventions, as realized in this study. The majority of the analyzed interventions used cognitive, behavioral, and psychoeducational components [27, 28], which highlights that there is room for improvement regarding the evidence base for psychodynamic family interventions such as the CHIMPS intervention. Our results also contrast with early small-scale evaluations of the CHIMPS intervention, which used a pre-post design and suggested relevantly improved mental health and health-related quality of life among CA after the intervention, while a waiting list control group showed no relevant changes [54, 55]. Coping with the parental mental illness, the scope and quality of interpersonal relationships, and family dynamics are assumed to be the intervention’s central modes of action and are relevant to CAs‘ mental health and health-related quality of life [15, 22, 23, 24, 25]. Therefore, it would not be appropriate to question the importance of these factors, but rather to examine whether participating family members improved in these variables. In fact, no relevant improvement was found in coping, family relationships, and social support, which might be a reason why the mental health and health-related quality of life of CA did not improve relevantly [56].

A cost-utility analysis was conducted by project partners at a participating center [57]. The intervention showed a high probability of being cost-effective compared with TAU. However, uncertainty around the estimates indicated that larger studies are needed to confirm these findings.

### Limitations

We would like to highlight a few areas for improvement from a psychotherapeutic perspective. The CHIMPS program is designed to last an average of eight sessions [35], but many families received less sessions. An analysis of the association between the number of therapy sessions and the therapy’s effectiveness is currently underway. Recent studies have demonstrated the effectiveness of short-term psychodynamic psychotherapy, but the average number of sessions in these studies was still well above eight [58, 59, 60]. There is evidence that short-term psychodynamic therapy can be insufficient for patients with complex or chronic mental illnesses, and that long-term psychodynamic therapy would be superior for these [61]. CHIMPS was designed as a low-threshold intervention which families should be able to participate in without much hassle — however, the scope of the program may not have been sufficient for all families. This point seems particularly relevant given that a considerable proportion of CA had a psychiatric diagnosis or psychopathological symptoms at baseline. It would be advisable to take a closer look at the therapeutic process within the scope of this study. Results should be evaluated regarding therapists‘ adherence to the manual, the therapeutic alliance, as well as the patients‘ assessment of treatment, satisfaction, and goal achievement.

Sandstrom et al. [29] emphasized the importance of very early interventions. CA in this sample were 9 years old on average, and it is possible that the intervention would be more effective for younger children.

Even though the CHIMPS program is deliberately designed to be transdiagnostic, a potentially wide range of diagnoses among mentally ill parents could have been challenging. However, almost all patients had a primary diagnosis in the F30-39 and F40-49 range (see Supplementary Material 2.b).

The general improvement in both groups over time could partially be explained by the diagnostic assessment including feedback at all measurement points, which may have had a quasi-therapeutic effect. Mangset et al. [62] showed that such assessment interviews can influence participants‘ outcomes. They identified four themes that participants associated with the assessments: They can serve as a safety net; enhance awareness and understanding; encourage participants to seek support; and allow the opportunity to vent disappointment. However, as a fifth theme, some participants did not feel any change as a result of the interviews alone. Participants in this study may have benefited psychologically from the interviews or been motivated to seek further help.

An alternative explanation for the apparent improvement is that potentially only participants who benefited from the study and were satisfied with it remained in the study, or that it was primarily stressed CA from stressed families who were lost to follow-up. If systematic loss to follow-up of this kind occurred, improvements over time would not be attributable to the intervention, but rather to a selective sample.

In general, recruitment revealed that this target group was very difficult to reach and also difficult to retain. Very few observations, especially for T4 (main measurement point of interest), were included, meaning that the study was underpowered and the meaningfulness of results is limited [63]. Retention of families in intervention studies must be taken into account when planning the study design. Family-based interventions can be disruptive for families with mentally ill parents, who already bear a heavy burden, and discussing the transgenerational risk of mental illness can be stressful for many parents and trigger feelings of guilt [28]. Briel et al. [64] found the following aspects of RCTs to be associated with poorer recruitment: narrower eligibility criteria, funding by investigators rather than the industry, a higher burden for patients and recruiters, outdated control interventions, and a later launch compared to RCTs investigating similar research questions. Our study was limited to a clinical sample (with children), but the eligibility criteria in terms of diagnostic spectrum and age of CA were relatively broad. Funding was external, the control condition was up to date (TAU), and — to the best of our knowledge — there is no abundance of family-oriented services for CHIMPS and their families. However, efforts may indeed have been too high for some of the families and recruiters. Families had to complete a relatively extensive questionnaire over a relatively long period of time. Recruiters who primarily acquired families through adult psychiatric wards, on the other hand, were potentially struggling to fit recruitment into the stressful daily routine of a clinic. Financial incentives, such as those paid in this study, have proven to be effective for recruitment and retention, but are ultimately one of many factors that influence participants‘ adherence [65].

The fact that the CG received TAU meant that families were allowed to seek psychiatric, psychotherapeutic, and other help, which made it more difficult to detect an effect and allowed a certain variability in the CG [66]. Additional treatments received by family members were not systematically recorded.

The low-threshold, low-frequency, and transdiagnostic nature of the intervention makes it easily accessible to many families. However, the exclusion of patients with acute severe psychiatric conditions implies that CA who are likely suffering most do not receive acute help through this program. Nonetheless, the CHIMPS program can provide families with coping strategies and resources for acute crises during periods when the mentally ill parent is more stable.

### Outlook

One approach to increase the intervention’s effectiveness is to align it with the specific psychosocial situation of families and CAs’ mental health [32, 67]. Some children may be more affected by parental mental illness than others. In addition, it may be useful to consider the initial symptom burden of mentally ill parents [14, 68] and family functionality [14] when applying interventions for CHIMPS [68]. Moltrecht et al. [27] and Lannes et al. [28] suggested that additional reminder sessions over time could improve the long-term outcomes of interventions, but emphasized that this aspect requires more systematic research.

It would be desirable for future studies to include a broader diagnostic spectrum to make statements about the transdiagnostic effectiveness of the CHIMPS intervention. The assessment of additional treatments should be systematized in the future so that it can be included as a covariate. In addition, the CHIMPS intervention should be compared with other family-based interventions or individual therapies for parents and/or children in an RCT. This would ensure that the CG is uniform and well-controlled.

## Conclusions

The primary results indicated that the CHIMPS intervention improved the status of CAs’ psychiatric diagnoses over time, but was not significantly superior to TAU. Therefore, the intervention likely needs to be further refined in order to effectively supplement the care situation for affected families, either by tailoring the intervention to families’ needs, or by extending the number of therapy sessions. Qualitative interviews with participating families could provide information about which needs of the families were met versus not met. The study emphasizes that the burdens associated with participation in a scientific study must be reduced for both families and recruiters. A more detailed analysis of the reasons why families with mentally ill parents leave an intervention and/or study at a given point in time will help to develop measures to increase participant satisfaction. In general, it is once again clear how important it is to improve care for CHIMPS, as about half of the CA in this sample had a psychiatric diagnosis at baseline, yet just under one eighth was in treatment at the time. The German healthcare system is clearly lacking adequate care in this area, and well-evaluated, effective family interventions are needed. Interdisciplinary structures should be created within the healthcare system to provide low-threshold access to support for CHIMPS and their families. We hope that this study will contribute to improving care for CHIMPS and inspire further research in this area.

## Supplementary Information

Below is the link to the electronic supplementary material.

Supplementary Material 1.

## Data Availability

The datasets used and/or analysed during the current study are available from Silke Wiegand-Grefe (swiegand-grefe@uke.de) on reasonable request. Furthermore, a statistical analysis plan can be requested from Silke Wiegand-Grefe. The corresponding study protocol has been published.

## Abbreviations

CA: Children and adolescents
CBCL: Child Behavior Checklist
CG: Control Group
CHIMPS: Children of mentally ill parents
FB-A: Allgemeiner Familienbogen [General Family Questionnaire
FKV: Freiburger Fragebogen zur Krankheitsverarbeitung [Freiburg Questionnaire on Coping
IG: Intervention Group
ITT: Intention-To-Treat
K-SADS-PL: Schedule for Affective Disorders and Schizophrenia for School- Age Children-Present and Lifetime Version
PP: Per-Protocol
RCT: Randomized Controlled Trial
TAU: Treatment-As-Usual
YSR: Youth Self Report
