# Supplementary material for: Effectiveness of a family-oriented intervention for children of mentally ill parents on children’s mental health and health-related quality of life: a prospective, rater-blinded, cluster-randomized controlled multicenter trial
Source: Child Adolesc Psychiatry Ment Health. 2026 Sep 25;20:135. doi: 10.1186/s13034-026-01174-6 (PMC13615611; doi:10.1186/s13034-026-01174-6)
Supplement: Supplementary file 1 [file 13034_2026_1174_MOESM1_ESM.zip › Supplementary Material_new/Supplementary Material 1_Methods_new.docx]

**Supplementary Material 1 – Data analysis**

**Supplementary Material 1.a** List of variables considered for test of imbalances at baseline measurement and used in

the imputation model

Variables of mentally ill parents rated by themselves or by physician, respectively

Age

Sex

Number of children

Marital status

Highest level of education

Highest professional qualification

PHQ-D

ICD-10 main diagnosis (rated by physician)

Number of comorbid ICD-10 diagnoses (rated by physician)

Variables of children rated by their mentally ill parent or by blinded rater, respectively

Age

Sex

ICD-10 main diagnosis (rated by blinded external rater using K-SADS-PL)

Number of comorbid ICD-10 diagnoses (rated by blinded external rater using K-SADS-PL)

K-SADS-PL = Schedule for Affective Disorders and Schizophrenia for School-Age Children-Present and Lifetime Version;

PHQ-D = Patient Health Questionnaire-Deutsch [German].

**Supplementary Material 1.b** Overview of primary and secondary endpoints considered for test of imbalances at baseline measurement used in the imputation model

| Outcome |  | Instrument | | Source | | |  |  |
| --- | --- | --- | --- | --- | --- | --- | --- | --- |
|  |  | **Questionnaire** | **Interview** | **Mentally ill parent** | **Second parent** | **Child**  **(> 10 years)** | | **External rater** |
| Demographics |  | Ad hoc items |  | x | x | x | |  |
| Diagnoses/  Psychiatric  symptomatology | Parents | Medical history |  |  |  |  | | x |
|  |  | BSI |  | x | x |  | |  |
|  |  | CGI |  |  |  |  | | x |
|  |  | GAF |  |  |  |  | | x |
|  | CA | CBCL | K-SADS-PL | x | x | x | |  |
|  |  | YSR |  |  |  | x | |  |
|  |  | SGKJ |  |  |  |  | | x |
| Health-related  quality of life | Parents | EQ-5D |  | x | x |  | |  |
|  | CA | KIDSCREEN-27 |  | x | x | x | |  |
| Family relations  and family functioning |  | FB-A |  | x | x | x | |  |
|  |  | GARF |  |  |  |  | | x |
|  |  | Ad hoc items |  | x | x | x | |  |
| Coping | Parents | FKV |  | x | x |  | |  |
| Social support |  | OSSS-3 |  | x | x | x | |  |

BSI = Brief Symptom Inventory; CA = Children and adolescents; CBCL = Child Behavior Checklist; CGI = Clinical Global Impression Scale; FB-A = Allgemeiner Familienbogen [General Family Questionnaire]; FKV = Freiburger Fragebogen zur Krankheitsverarbeitung [Freiburg Questionnaire on Coping]; GAF = Global Assessment of Functioning; GARF = Global Assessment of Relational Functioning; K-SADS-PL = Schedule for Affective Disorders and Schizophrenia for School-Age Children-Present and Lifetime Version; OSSS-3 = Oslo Social Support Scale; SGKJ = Skala zur Gesamtbeurteilung von Kindern und Jugendlichen [Children Global Assessment Scale]; YSR = Youth Self Report.

**Supplementary Material 1.c** Imputation method

We used the Direct Maximum Likelihood as statistical estimation procedure in the primary endpoint analysis, which results in unbiased estimations under the Missing at Random assumption. In case of missing baseline values and the need to include unbalanced baseline variables in the primary analysis model, these values were imputed by an Expectation-Maximization algorithm with all relevant baseline variables and the valid primary endpoints at all measurement points as independent variables. Missing follow-up values were imputed by two different methods, i. e., Multiple Imputation and Last Observation Carried Forward. For Multiple Imputation, all variables of the primary endpoint analysis and all relevant baseline variables were included in the imputation model. One hundred imputations were performed, and results were combined using Rubin‘s Rules. The relevant baseline variables were the variables in Supplementary Material 1.a and the baseline measurement of primary and secondary endpoints (see Supplementary Material 1.b).
