# Supplementary material for: Effectiveness of a family-oriented intervention for children of mentally ill parents on children’s mental health and health-related quality of life: a prospective, rater-blinded, cluster-randomized controlled multicenter trial
Source: Child Adolesc Psychiatry Ment Health. 2026 Sep 25;20:135. doi: 10.1186/s13034-026-01174-6 (PMC13615611; doi:10.1186/s13034-026-01174-6)
Supplement: Supplementary file 1 [file 13034_2026_1174_MOESM1_ESM.zip › Supplementary Material_new/Supplementary Material 2_Results_new.docx]

**Supplementary Material 2 – Results**

**Supplementary Material 2.a** Medical status and history of mentally ill parents

|  | **Total (*n* = 214)** | **IG (*n* = 108)** | **CG (*n* = 106)** |
| --- | --- | --- | --- |
| **Physical illness** |  |  |  |
| Information missing | 11/214 (5.1%) | 5/108 (4.6%) | 6/106 (5.7%) |
| Currently healthy | 101/203 (49.8%)) | 53/103 (51.5%) | 48/100 (48.0%) |
| Currently ill | 102/203 (50.2%) | 50/103 (48.5%) | 52/100 (52.0%) |
| **Mental illness** |  |  |  |
| Information missing | 12/214 (5.6%) | 7/108 (6.5%) | 5/106 (4.7%) |
| Currently healthy | 3/202 (1.5%) | 1/101 (1.0%) | 2/101 (2.0%) |
| Currently ill | 195/202 (96.5%) | 96/101 (95.0%) | 99/101 (98.0%) |
| Previously ill | 4/202 (2.0%) | 4/101 (4.0%) | 0/101 (0.0%) |
| **Past inpatient treatment for mental illness** |  |  |  |
| Information missing | 8/214 (3.7%) | 5/108 (4.6%) | 3/106 (2.8%) |
| No | 52/206 (25.2%) | 29/103 (28.2%) | 23/103 (22.3%) |
| Yes | 154/206 (74.8%) | 74/103 (71.8%) | 80/103 (77.7%) |
| **Current outpatient treatment for mental illness** |  |  |  |
| Information missing | 9/214 (4.2%) | 5/108 (4.6%) | 4/106 (3.8%) |
| No | 66/205 (32.2%) | 30/103 (29.1%) | 36/102 (35.3%) |
| Yes | 139/205 (67.8%) | 73/103 (70.9%) | 66/102 (64.7%) |

CG = Control Group; IG = Intervention Group.

**Supplementary Material 2.b** Frequency of specific ICD-10 diagnoses of mentally ill parents

|  | **Total (*n* = 214)** | **IG (*n* = 108)** | **CG (*n* = 106)** |
| --- | --- | --- | --- |
| **ICD-10 main diagnosis** |  |  |  |
| Information missing | 0/214 (0.0%) | 0/108 (0.0%) | 0/106 (0.0%) |
| F10-F19 | 3/214 (1.4%) | 1/108 (0.9%) | 2/106 (1.9%) |
| F20-F29 | 12/214 (5.6%) | 7/108 (6.5%) | 5/106 (4.7%) |
| F30-F39 | 118/214 (55.1%) | 61/108 (56.5%) | 57/106 (53.8%) |
| F40-F49 | 25/214 (11.7%) | 10/108 (9.3%) | 15/106 (14.2%) |
| F50-F59 | 0/214 (0.0%) | 0/108 (0.0%) | 0/106 (24.5%) |
| F60-F69 | 0/214 (0.0%) | 0/108 (0.0%) | 0/106 (0.0%) |
| F70-F79 | 0/214 (0.0%) | 0/108 (0.0%) | 0/106 (0.0%) |
| F80-F89 | 0/214 (0.0%) | 0/108 (0.0%) | 0/106 (0.0%) |
| F90-F99 | 1/214 (0.5%) | 0/108 (0.0%) | 1/106 (0.9%) |
| **ICD-10 second diagnosis** |  |  |  |
| Information missing | 128/214 (59.8%) | 62/108 (57.4%) | 66/106 (62.3%) |
| F10-F19 | 4/86 (4.7%) | 4/46 (8.7%) | 0/40 (0.0%) |
| F20-F29 | 1/86 (1.2%) | 0/46 (0.0%) | 1/40 (2.5%) |
| F30-F39 | 41/86 (47.7%) | 21/46 (45.7%) | 20/40 (50.0%) |
| F40-F49 | 29/86 (33.7%) | 13/46 (28.3%) | 16/40 (40.0%) |
| F50-F59 | 7/86 (8.1%) | 5/46 (10.9%) | 2/40 (5.0%) |
| F60-F69 | 1/86 (1.2%) | 1/46 (2.2%) | 0/40 (0.0%) |
| F70-F79 | 1/86 (1.2%) | 1/46 (2.2%) | 0/40 (0.0%) |
| F80-F89 | 0/86 (0.0%) | 0/46 (0.0%) | 0/40 (0.0%) |
| F90-F99 | 2/86 (2.3%) | 1/46 (2.2%) | 1/40 (2.5%) |
| **ICD-10 third diagnosis** |  |  |  |
| Information missing | 183/214 (85.5%) | 96/108 (88.9%) | 87/106 (82.1%) |
| F10-F19 | 7/31 (22.6%) | 1/12 (8.3%) | 6/19 (31.6%) |
| F20-F29 | 0/31 (0.0%) | 0/12 (0.0%) | 0/19 (0.0%) |
| F30-F39 | 7/31 (22.6%) | 3/12 (25.0%) | 4/19 (21.1%) |
| F40-F49 | 10/31 (32.3%) | 6/12 (50.0%) | 4/19 (21.1%) |
| F50-F59 | 4/31 (12.9%) | 2/12 (16.7%) | 2/19 (10.5%) |
| F60-F69 | 0/31 (0.0%) | 0/12 (0.0%) | 0/19 (0.0%) |
| F70-F79 | 0/31 (0.0%) | 0/12 (0.0%) | 0/19 (0.0%) |
| F80-F89 | 0/31 (0.0%) | 0/12 (0.0%) | 0/19 (0.0%) |
| F90-F99 | 3/31 (9.7%) | 0/12 (0.0%) | 3/19 (15.8%) |
| **ICD-10 fourth diagnosis** |  |  |  |
| Information missing | 204/214 (95.3%) | 106/108 (98.1%) | 98/106 (92.5%) |
| F10-F19 | 2/10 (20.0%) | 0/2 (0.0%) | 2/8 (25.0%) |
| F20-F29 | 0/10 (0.0%) | 0/2 (0.0%) | 0/8 (0.0%) |
| F30-F39 | 2/10 (20.0%) | 0/2 (0.0%) | 2/8 (25.0%) |
| F40-F49 | 2/10 (20.0%) | 1/2 (50.0%) | 1/8 (12.5%) |
| F50-F59 | 2/10 (20.0%) | 1/2 (50.0%) | 1/8 (12.5%) |
| F60-F69 | 0/10 (0.0%) | 0/2 (0.0%) | 0/8 (0.0%) |
| F70-F79 | 0/10 (0.0%) | 0/2 (0.0%) | 0/8 (0.0%) |
| F80-F89 | 1/10 (10.0%) | 0/2 (0.0%) | 1/8 (12.5%) |
| F90-F99 | 1/10 (10.0%) | 0/2 (0.0%) | 1/8 (12.5%) |

CG = Control Group; IG = Intervention Group.

**Supplementary Material 2.c** Frequency of quantities of mentally ill parents‘ ICD-10 diagnoses per person

|  | **Total (*n* = 214)** | **IG (*n* = 108)** | **CG (*n* = 106)** |
| --- | --- | --- | --- |
| **Quantity of ICD-10 diagnoses** |  |  |  |
| Information missing | 0/214 (0.0%) | 0/108 (0.0%) | 0/106 (0.0%) |
| 1 | 128/214 (59.8%) | 62/108 (57.4%) | 66/106 (62.3%) |
| 2 | 55/214 (25.7%) | 34/108 (31.5%) | 21/106 (19.8%) |
| 3 | 21/214 (9.8%) | 10/108 (9.3%) | 11/106 (10.4%) |
| 4 | 10/214 (4.7%) | 2/108 (1.9%) | 8/106 (7.5%) |

CG = Control Group; IG = Intervention Group.

**Supplementary Material 2.d** Frequency of specific ICD-10 diagnoses of CA

|  | **Total (*n* = 337)** | **IG (*n* = 170)** | **CG (*n* = 167)** |
| --- | --- | --- | --- |
| **No diagnosis** | 134/337 (39.8%) | 70/170 (41.2%) | 64/167 (38.3%) |
| **ICD-10 main diagnosis** |  |  |  |
| Information missing | 33/203 (16.3%) | 15/100 (15.0%) | 18/103 (17.5%) |
| F10-F19 | 0/170 (0.0%) | 0/85 (0.0%) | 0/85 (0.0%) |
| F20-F29 | 0/170 (0.0%) | 0/85 (0.0%) | 0/85 (0.0%) |
| F30-F39 | 43/170 (25.3%) | 22/85 (25.9%) | 21/85 (24.7%) |
| F40-F49 | 64/170 (37.6%) | 29/85 (34.1%) | 35/85 (41.2%) |
| F50-F59 | 1/170 (0.6%) | 1/85 (1.2%) | 0/85 (0.0%) |
| F60-F69 | 0/170 (0.0%) | 0/85 (0.0%) | 0/85 (0.0%) |
| F70-F79 | 0/170 (0.0%) | 0/85 (0.0%) | 0/85 (0.0%) |
| F80-F89 | 0/170 (0.0%) | 0/85 (0.0%) | 0/85 (0.0%) |
| F90-F99 | 62/170 (36.5%) | 33/85 (38.8%) | 29/85 (34.1%) |
| Z | 0/170 (0.0%) | 0/85 (0.0%) | 0/85 (0.0%) |
| T | 0/170 (0.0%) | 0/85 (0.0%) | 0/85 (0.0%) |
| **ICD-10 second diagnosis** |  |  |  |
| Information missing | 115/203 (56.7%) | 60/100 (60.0%) | 55/103 (53.4%) |
| F10-F19 | 0/88 (0.0%) | 0/40 (0.0%) | 0/48 (0.0%) |
| F20-F29 | 0/88 (0.0%) | 0/40 (0.0%) | 0/48 (0.0%) |
| F30-F39 | 3/88 (3.4%) | 3/40 (7.5%) | 0/48 (0.0%) |
| F40-F49 | 40/88 (45.5%) | 16/40 (40.0%) | 24/48 (50.0%) |
| F50-F59 | 1/88 (1.1%) | 0/40 (0.0%) | 1/48 (2.1%) |
| F60-F69 | 0/88 (0.0%) | 0/40 (0.0%) | 0/48 (0.0%) |
| F70-F79 | 0/88 (0.0%) | 0/40 (0.0%) | 0/48 (0.0%) |
| F80-F89 | 1/88 (1.1%) | 1/40 (2.5%) | 0/48 (0.0%) |
| F90-F99 | 42/88 (47.7%) | 20/40 (50.0%) | 22/48 (45.8%) |
| Z | 1/88 (1.1%) | 0/40 (0.0%) | 1/48 (2.1%) |
| T | 0/88 (0.0%) | 0/40 (0.0%) | 0/48 (0.0%) |
| **ICD-10 third diagnosis** |  |  |  |
| Information missing | 166/203 (81.8%) | 82/100 (82.0%) | 84/103 (81.6%) |
| F10-F19 | 2/37 (5.4%) | 1/18 (5.6%) | 1/19 (5.3%) |
| F20-F29 | 0/37 (0.0%) | 0/18 (0.0%) | 0/19 (0.0%) |
| F30-F39 | 1/37 (2.7%) | 0/18 (0.0%) | 1/19 (5.3%) |
| F40-F49 | 11/37 (29.7%) | 4/18 (22.2%) | 7/19 (36.8%) |
| F50-F59 | 0/37 (0.0%) | 0/18 (0.0%) | 0/19 (0.0%) |
| F60-F69 | 0/37 (0.0%) | 0/18 (0.0%) | 0/19 (0.0%) |
| F70-F79 | 0/37 (0.0%) | 0/18 (0.0%) | 0/19 (0.0%) |
| F80-F89 | 0/37 (0.0%) | 0/18 (0.0%) | 0/19 (0.0%) |
| F90-F99 | 21/37 (56.8%) | 12/18 (66.7%) | 9/19 (47.4%) |
| Z | 0/37 (0.0%) | 0/18 (0.0%) | 0/19 (0.0%) |
| T | 2/37 (5.4%) | 1/18 (5.6%) | 1/19 (5.3%) |
| **ICD-10 fourth diagnosis** |  |  |  |
| Information missing | 188/203 (92.6%) | 95/100 (95.0%) | 93/103 (90.3%) |
| F10-F19 | 0/15 (0.0%) | 0/5 (0.0%) | 0/10 (0.0%) |
| F20-F29 | 0/15 (0.0%) | 0/5 (0.0%) | 0/10 (0.0%) |
| F30-F39 | 5/15 (33.3%) | 1/5 (20.0%) | 4/10 (40.0%) |
| F40-F49 | 5/15 (33.3%) | 3/5 (60.0%) | 2/10 (20.0%) |
| F50-F59 | 0/15 (0.0%) | 0/5 (0.0%) | 0/10 (0.0%) |
| F60-F69 | 0/15 (0.0%) | 0/5 (0.0%) | 0/10 (0.0%) |
| F70-F79 | 0/15 (0.0%) | 0/5 (0.0%) | 0/10 (0.0%) |
| F80-F89 | 0/15 (0.0%) | 0/5 (0.0%) | 0/10 (0.0%) |
| F90-F99 | 5/15 (33.3%) | 1/5 (20.0%) | 4/10 (40.0%) |
| Z | 0/15 (0.0%) | 0/5 (0.0%) | 0/10 (0.0%) |
| T | 0/15 (0.0%) | 0/5 (0.0%) | 0/10 (0.0%) |

CA = children and adolescents; CG = Control Group; IG = Intervention Group.

**Supplementary Material 2.e** Frequency of quantities of CAs‘ ICD-10 diagnoses per child/adolescent

|  | **Total (*n* = 214)** | **IG (*n* = 108)** | **CG (*n* = 106)** |
| --- | --- | --- | --- |
| **Quantity of ICD-10 diagnoses** |  |  |  |
| Information missing | 33/337 (9.8%) | 15/170 (8.8%) | 18/167 (10.8%) |
| 1 | 134/304 (44.1%) | 70/155 (45.2%) | 64/149 (43.0%) |
| 2 | 82/304 (27.0%) | 45/155 (29.0%) | 37/149 (24.8%) |
| 3 | 22/304 (7.2%) | 13/155 (8.4%) | 9/149 (6.0%) |
| 4 | 15/304 (4.9%) | 5/155 (3.2%) | 10/149 (6.7%) |

CA = children and adolescents; CG = Control Group; IG = Intervention Group.

**Supplementary Material 2.f** KIDSCREEN-10 (proxy) total raw score in IG and CG in the ITT2 population over time. Please note: The inconsistency between the left and middle panel at T4 is due to small sample size at T4 and a high proportion of CA with missing data at T1. CA = children and adolescents; CG = Control Group; IG = Intervention Group; ITT = Intention-To-Treat.

**Supplementary Material 2.g** Results of secondary endpoint analysis on KIDSCREEN-10 (proxy) total raw score in the ITT2 population

|  | **IG** | | | **CG** | | | **IG versus CG** | | |  |
| --- | --- | --- | --- | --- | --- | --- | --- | --- | --- | --- |
| **Outcome** | ***n*** | **obs. *M* (*SD*)** | **adj. *M* (95% CI)** | ***n*** | **obs. *M* (*SD*)** | **adj. *M***  **(95% CI)** | **adj. *M* diff.**  **(95% CI)** | ***p*-value** | ***p*-value**  **interaction (treatment group-time)** | ***n*** |
| **KIDSCREEN-10** |  |  |  |  |  |  | 0.09  (-0.35; 0.53) | 0.6858 | 0.8790 | 91 |
| **T1** | 49 | 0.52 (0.79) |  | 64 | 0.46 (0.78) |  |  |  |  |  |
| **T2-T1** | 22 | 0.22 (0.67) | 0.34  (-0.00; 0.68) | 33 | 0.44 (1.03) | 0.25  (-0.04; 0.53) |  |  |  |  |
| **T3-T1** | 15 | 0.53 (1.01) | 0.69  (0.34; 1.05) | 19 | 0.42 (0.90) | 0.60  (0.28; 0.92) |  |  |  |  |
| **T4-T1** | 0 | NaN (NA) | 0.19  (-0.59; 0.96) | 3 | 0.41 (0.57) | 0.10  (-0.63; 0.83) |  |  |  |  |

Adj. *M* = adjusted Mean; CG = Control Group; CI = Confidence Interval; diff. = difference; IG = Intervention Group; ITT = Intention-To-Treat; obs. *M* = observed Mean; NaN = Not a Number; NA = Not Available; *SD* = Standard Deviation.

**Supplementary Material 2.h** KIDSCREEN-10 (proxy) total raw score in IG and CG in the ITT3 population over time. Please note: The inconsistency between the left and middle panel at T4 is due to small sample size at T4 and a high proportion of CA with missing data at T1. CA = children and adolescents; CG = Control Group; IG = Intervention Group; ITT = Intention-To-Treat.

**Supplementary Material 2.i** Results of secondary endpoint analysis on KIDSCREEN-10 (proxy) total raw score in the ITT3 population

|  | **IG** | | | **CG** | | | **IG versus CG** | | |  |
| --- | --- | --- | --- | --- | --- | --- | --- | --- | --- | --- |
| **Outcome** | ***n*** | **obs. *M* (*SD*)** | **adj. *M* (95% CI)** | ***n*** | **obs. *M* (*SD*)** | **adj. *M***  **(95% CI)** | **adj. *M* diff.**  **(95% CI)** | ***p*-value** | ***p*-value**  **interaction (treatment group-time)** | ***n*** |
| **KIDSCREEN-10** |  |  |  |  |  |  | 0.24  (-0.18; 0.65) | 0.2522 | 0.1736 | 93 |
| **T1** | 48 | 0.81 (1.05) |  | 50 | 0.87 (1.08) |  |  |  |  |  |
| **T2-T1** | 22 | -0.10 (0.98) | 0.08  (-0.24; 0.40) | 29 | 0.12 (0.99) | -0.15  (-0.44; 0.13) |  |  |  |  |
| **T3-T1** | 17 | 0.49 (1.06) | 0.47  (0.14; 0.80) | 22 | 0.12 (1.06) | 0.23  (-0.08; 0.54) |  |  |  |  |
| **T4-T1** | 1 | -1.21 (NA) | -0.05  (-0.75; 0.66) | 3 | 0.41 (0.57) | -0.28  (-0.96; 0.39) |  |  |  |  |

Adj. *M* = adjusted Mean; CG = Control Group; CI = Confidence Interval; diff. = difference; IG = Intervention Group; ITT = Intention-To-Treat; obs. *M* = observed Mean; NA = Not Available; *SD* = Standard Deviation.

**Supplementary Material 2.j** YSR total raw score in IG and CG in the ITT1 population over time. Please note: The inconsistency between the left and middle panel at T4 is due to small sample size at T4 and a high proportion of CA with missing data at T1. CA = children and adolescents; CG = Control Group; IG = Intervention Group; ITT = Intention-To-Treat; YSR = Youth Self Report.

**Supplementary Material 2.k** Results of secondary endpoint analysis on YSR total raw score in the ITT1 population

|  | **IG** | | | **CG** | | | **IG versus CG** | | |  |
| --- | --- | --- | --- | --- | --- | --- | --- | --- | --- | --- |
| **Outcome** | ***n*** | **obs. *M***  **(*SD*)** | **adj. *M***  **(95% CI)** | ***n*** | **obs. *M***  **(*SD*)** | **adj. *M***  **(95% CI)** | ***M* diff. (95% CI)** | ***p*-value** | ***p*-value interaction (treatment group-time)** | ***n*** |
| **YSR** |  |  |  |  |  |  | -6.51  (-13.90;  0.88) | 0.0829 | 0.3538 | 122 |
| **T1** | 62 | 66.66  (21.15) |  | 73 | 66.85  (19.59) |  |  |  |  |  |
| **T2-T1** | 25 | -9.08  (21.20) | -12.82  (-21.22;  -4.43) | 39 | -11.13 (13.26) | -6.31  (-13.43; 0.81) |  |  |  |  |
| **T3-T1** | 17 | -17.24 (16.82) | -14.21  (-23.26;  -5.15) | 28 | -8.86  (17.52) | -7.70  (-15.42; 0.03) |  |  |  |  |
| **T4-T1** | 8 | -19.75 (17.29) | -16.20  (-26.05;  -6.35) | 16 | -11.94 (17.23) | -9.69  (-18.24;  -1.14) |  |  |  |  |

Adj. *M* = adjusted Mean; CG = Control Group; CI = Confidence Interval; diff. = difference; IG = Intervention Group; ITT = Intention-To-Treat; obs. *M* = observed Mean; *SD* = Standard Deviation; YSR = Youth Self Report.

**Supplementary Material 2.l** KIDSCREEN-10 (self) total raw score in IG and CG in the ITT1 population over time. Please note: The inconsistency between the left and middle panel at T4 is due to small sample size at T4 and a high proportion of CA with missing data at T1. CA = children and adolescents; CG = Control Group; IG = Intervention Group; ITT = Intention-To-Treat.

**Supplementary Material 2.m** Results of secondary endpoint analysis on KIDSCREEN-10 (self) total raw score in the ITT1 population

|  | **IG** | | | **CG** | | | **IG versus CG** | | |  |
| --- | --- | --- | --- | --- | --- | --- | --- | --- | --- | --- |
| **Outcome** | ***n*** | **obs. *M***  **(*SD*)** | **adj. *M***  **(95% CI)** | ***n*** | **obs. *M***  **(*SD*)** | **adj. *M***  **(95% CI)** | ***M* diff. (95% CI)** | ***p*-value** | ***p*-value interaction (treatment group-time)** | ***n*** |
| **KIDSCREEN-10** |  |  |  |  |  |  | 0.36  (-0.03; 0.74) | 0.0674 | 0.5089 | 105 |
| **T1** | 59 | 0.85 (0.94) |  | 69 | 1.02 (1.12) |  |  |  |  |  |
| **T2-T1** | 22 | 0.44 (0.70) | 0.55  (0.12; 0.97) | 33 | 0.31 (0.84) | 0.19  (-0.16; 0.53) |  |  |  |  |
| **T3-T1** | 16 | 0.45 (0.65) | 0.35  (-0.11; 0.82) | 22 | -0.07 (1.03) | -0.00  (-0.40; 0.39) |  |  |  |  |
| **T4-T1** | 7 | 0.25 (0.88) | 0.30  (-0.23; 0.83) | 12 | 0.11 (0.78) | -0.06  (-0.52; 0.41) |  |  |  |  |

Adj. *M* = adjusted Mean; CG = Control Group; CI = Confidence Interval; diff. = difference; IG = Intervention Group; ITT = Intention-To-Treat; obs. *M* = observed Mean; *SD* = Standard Deviation.

**Supplementary Material 2.n** KIDSCREEN-10 (self) total raw score in IG and CG in the ITT2 population over time. Please note: The inconsistency between the left and middle panel at T4 is due to small sample size at T4 and a high proportion of CA with missing data at T1. CA = children and adolescents; CG = Control Group; IG = Intervention Group; ITT = Intention-To-Treat.

**Supplementary Material 2.o** Results of secondary endpoint analysis on KIDSCREEN-10 (self) total raw score in the ITT2 population

|  | **IG** | | | **CG** | | | **IG versus CG** | | |  |
| --- | --- | --- | --- | --- | --- | --- | --- | --- | --- | --- |
| **Outcome** | ***n*** | **obs. *M* (*SD*)** | **adj. *M* (95% CI)** | ***n*** | **obs. *M* (*SD*)** | **adj. *M***  **(95% CI)** | **adj. *M* diff.**  **(95% CI)** | ***p*-value** | ***p*-value**  **interaction (treatment group-time)** | ***n*** |
| **KIDSCREEN-10** |  |  |  |  |  |  | 0.28  (-0.29; 0.85) | 0.3153 | 0.9028 | 49 |
| **T1** | 20 | 0.55 (0.74) |  | 36 | 0.69 (1.08) |  |  |  |  |  |
| **T2-T1** | 6 | 0.43 (0.74) | 0.62  (0.10; 1.14) | 20 | 0.30 (0.85) | 0.34  (0.03; 0.65) |  |  |  |  |
| **T3-T1** | 3 | 0.00 (0.49) | 0.27  (-0.29; 0.83) | 12 | 0.02 (1.06) | -0.01  (-0.36; 0.35) |  |  |  |  |
| **T4-T1** | 1 | 0.22 (NA) | 0.15  (-0.46; 0.76) | 8 | 0.11 (0.87) | -0.13  (-0.59; 0.33) |  |  |  |  |

Adj. *M* = adjusted Mean; CG = Control Group; CI = Confidence Interval; diff. = difference; IG = Intervention Group; ITT = Intention-To-Treat; obs. *M* = observed Mean; NA = Not Available; *SD* = Standard Deviation.

**Supplementary Material 2.p** KIDSCREEN-10 (self) total raw score in IG and CG in the ITT3 population over time. Please note: The inconsistency between the left and middle panel at T4 is due to small sample size at T4 and a high proportion of CA with missing data at T1. CA = children and adolescents; CG = Control Group; IG = Intervention Group; ITT = Intention-To-Treat.

**Supplementary Material 2.q** Results of secondary endpoint analysis on KIDSCREEN-10 (self) total raw score in the ITT3 population

|  | **IG** | | | **CG** | | | **IG versus CG** | | |  |
| --- | --- | --- | --- | --- | --- | --- | --- | --- | --- | --- |
| **Outcome** | ***n*** | **obs. *M* (*SD*)** | **adj. *M* (95% CI)** | ***n*** | **obs. *M* (*SD*)** | **adj. *M***  **(95% CI)** | **adj. *M* diff.**  **(95% CI)** | ***p*-value** | ***p*-value**  **interaction (treatment group-time)** | ***n*** |
| **KIDSCREEN-10** |  |  |  |  |  |  | 0.47  (-0.54; 1.47) | 0.2433 | 0.3479 | 39 |
| **T1** | 17 | 0.39 (0.78) |  | 25 | 0.63 (0.84) |  |  |  |  |  |
| **T2-T1** | 6 | 0.53 (0.99) | 0.68  (-0.05; 1.40) | 14 | 0.07 (0.53) | 0.21  (-0.26; 0.68) |  |  |  |  |
| **T3-T1** | 4 | 0.78 (0.71) | 0.59  (-0.15; 1.34) | 10 | 0.07 (0.87) | 0.13  (-0.33; 0.59) |  |  |  |  |
| **T4-T1** | 4 | 0.60 (0.92) | 0.64  (-0.06; 1.34) | 6 | 0.31 (0.84) | 0.17  (-0.36; 0.70) |  |  |  |  |

Adj. *M* = adjusted Mean; CG = Control Group; CI = Confidence Interval; diff. = difference; IG = Intervention Group; ITT = Intention-To-Treat; obs. *M* = observed Mean; *SD* = Standard Deviation.

**Supplementary Material 2.r** Results of sensitivity analysis on K-SADS-PL diagnoses in the PP3 population

|  | **IG** | **CG** | **IG versus CG** | | |  |
| --- | --- | --- | --- | --- | --- | --- |
| **Outcome** | **events/total (%)** | **events/total (%)** | **adj. OR (95% CI)** | ***p*-value** | ***p*-value**  **interaction (treatment group-time)** | ***n*** |
| **K-SADS-PL**  **diagnosis** |  |  | 0.47 (0.21; 1.05) | 0.0646 | 0.9934 | 164 |
| **T2** | 13/29 (44.83) | 24/38 (63.16) |  |  |  |  |
| **T3** | 12/23 (52.17) | 19/28 (67.86) |  |  |  |  |
| **T4** | 8/21 (38.10) | 15/26 (57.69) |  |  |  |  |

Adj. OR = adjusted Odds Ratio; CG = Control Group; CI = Confidence Interval; IG = Intervention Group; K-SADS-PL = Schedule for Affective Disorders and Schizophrenia for School-Age Children-Present and Lifetime Version; PP = Per-Protocol.

**Supplementary Material 2.s** Results of sensitivity analysis on K-SADS-PL diagnoses in the ITT3 population with imputed data

| **Method** | **OR (95%CI)** | ***p*-value** | ***p*-value interaction**  **(treatment group-time)** |
| --- | --- | --- | --- |
| **Multiple**  **Imputation** | 0.674 (0.329; 1.381) | 0.282 | 0.964 |
| **LOCF** | 0.488 (0.168; 1.423) | 0.189 | 0.844 |

CI = Confidence Interval; LOCF = Last Observation Carried Forward.

**Supplementary Material 2.t** Results of sensitivity analysis on K-SADS-PL diagnoses in the ITT3 population with outcome *Confirmed diagnosis* or *Near-diagnosis*

|  | **IG** | **CG** | **IG versus CG** | | |  |
| --- | --- | --- | --- | --- | --- | --- |
| **Outcome** | **events/total (%)** | **events/total (%)** | **adj. OR (95% CI)** | ***p*-value** | ***p*-value**  **interaction (treatment group-time)** | ***n*** |
| **K-SADS-PL**  **diagnosis** |  |  | 0.71 (0.30; 1.68) | 0.4417 | 0.5611 | 189 |
| **T2** | 26/42 (61.90) | 28/38 (73.68) |  |  |  |  |
| **T3** | 24/35 (68.57) | 25/28 (89.29) |  |  |  |  |
| **T4** | 17/29 (58.62) | 18/26 (69.23) |  |  |  |  |

Adj. OR = adjusted Odds Ratio; CG = Control Group; CI = Confidence Interval; IG = Intervention Group; ITT = Intention-To-Treat; K-SADS-PL = Schedule for Affective Disorders and Schizophrenia for School-Age Children-Present and Lifetime Version.

**Supplementary Material 2.u** Results of subgroup analyses on K-SADS-PL diagnoses in the ITT3 population with subgroups recruitment center, type of clinic, CAs' age, and CAs' sex

|  | | | **IG** | **CG** | **IG versus CG** |  |
| --- | --- | --- | --- | --- | --- | --- |
| **Subgroup** | **Subgroup level** | **Time** | **events/total (%)** | **events/total (%)** | **adj. OR (95% CI)** | ***p*-value**  **interaction group-**  **subgroup** |
| **Recruitment center** |  |  |  |  |  | 0.1965 |
|  | BE | T2 | 3/4 (75.00) | 3/6 (50.00) | 0.44 (0.05; 3.49) |  |
|  | BE | T3 | 2/3 (66.67) | 2/5 (40.00) | 0.44 (0.05; 3.49) |  |
|  | BE | T4 | 1/2 (50.00) | 1/5 (20.00) | 0.44 (0.05; 3.49) |  |
|  | GP | T3 | 1/2 (50.00) | NA/NA (NA) | NA (NA; NA) |  |
|  | GP | T4 | 2/3 (66.67) | NA/NA (NA) | NA (NA; NA) |  |
|  | HH | T2 | 11/16 (68.75) | 8/18 (44.44) | 0.52 (0.18; 1.53) |  |
|  | HH | T3 | 9/12 (75.00) | 9/17 (52.94) | 0.52 (0.18; 1.53) |  |
|  | HH | T4 | 7/12 (58.33) | 5/16 (31.25) | 0.52 (0.18; 1.53) |  |
|  | LZ | T2 | 1/5 (20.00) | 4/9 (44.44) | 3.71 (0.57; 24.13) |  |
|  | LZ | T3 | 1/5 (20.00) | 3/7 (42.86) | 3.71 (0.57; 24.13) |  |
|  | LZ | T4 | 2/5 (40.00) | 2/5 (40.00) | 3.71 (0.57; 24.13) |  |
|  | UG | T2 | 7/10 (70.00) | 4/8 (50.00) | 0.17 (0.03; 0.94) |  |
|  | UG | T3 | 5/5 (100.00) | 2/5 (40.00) | 0.17 (0.03; 0.94) |  |
|  | UG | T4 | 3/4 (75.00) | 1/3 (33.33) | 0.17 (0.03; 0.94) |  |
|  | WR | T2 | 2/2 (100.00) | NA/NA (NA) | 0.00 (0.00; inf.) |  |
|  | WR | T3 | 1/1 (100.00) | 1/1 (100.00) | 0.00 (0.00; inf.) |  |
| **Type of clinic** |  |  |  |  |  | 0.8547 |
|  | University clinic | T2 | 22/35 (62.86) | 19/41 (46.34) | 0.53 (0.25; 1.17) |  |
|  | University clinic | T3 | 17/25 (68.00) | 16/34 (47.06) | 0.53 (0.25; 1.17) |  |
|  | University clinic | T4 | 13/23 (56.52) | 9/29 (31.03) | 0.53 (0.25; 1.17) |  |
|  | Communal clinic | T2 | 2/3 (66.67) | NA/NA (NA) | 0.37 (0.01; 15.40) |  |
|  | Communal clinic | T3 | 2/3 (66.67) | 1/1 (100.00) | 0.37 (0.01; 15.40) |  |
|  | Communal clinic | T4 | 2/3 (66.67) | NA/NA (NA) | 0.37 (0.01; 15.40) |  |
| **CAs’ age** |  |  |  |  |  | 0.8371 |
|  | 3-13 years | T2 | 16/27 (59.26) | 16/36 (44.44) | 0.58 (0.25; 1.35) |  |
|  | 3-13 years | T3 | 12/17 (70.59) | 15/31 (48.39) | 0.58 (0.25; 1.35) |  |
|  | 3-13 years | T4 | 9/17 (52.94) | 8/26 (30.77) | 0.58 (0.25; 1.35) |  |
|  | 14-19 years | T2 | 8/11 (72.73) | 3/6 (50.00) | 0.47 (0.07; 3.28) |  |
|  | 14-19 years | T3 | 7/11 (63.64) | 2/4 (50.00) | 0.47 (0.07; 3.28) |  |
|  | 14-19 years | T4 | 6/9 (66.67) | 1/3 (33.33) | 0.47 (0.07; 3.28) |  |
| **CAs’ sex** |  |  |  |  |  | 0.8609 |
|  | Female | T2 | 12/19 (63.16) | 8/20 (40.00) | 0.48 (0.16; 1.44) |  |
|  | Female | T3 | 9/14 (64.29) | 8/16 (50.00) | 0.48 (0.16; 1.44) |  |
|  | Female | T4 | 8/13 (61.54) | 2/13 (15.38) | 0.48 (0.16; 1.44) |  |
|  | Male | T2 | 12/19 (63.16) | 11/22 (50.00) | 0.55 (0.20; 1.51) |  |
|  | Male | T3 | 10/14 (71.43) | 9/19 (47.37) | 0.55 (0.20; 1.51) |  |
|  | Male | T4 | 7/13 (53.85) | 7/16 (43.75) | 0.55 (0.20; 1.51) |  |

Adj. OR = adjusted Odds Ratio; BE = Berlin; CA = Children and adolescents; CG = Control Group; CI = Confidence Interval; GP = Gütersloh-Paderborn; HH = Hansestadt Hamburg; IG = Intervention Group; inf. = infinity; LZ = Leipzig; NA = Not Available; UG = Ulm-Günzburg; WR = Wiesbaden Rheingau.
